# The use and effects of telemedicine on complementary, alternative, and integrative medicine practices: a scoping review

**DOI:** 10.1186/s12906-023-04100-x

**Published:** 2023-08-02

**Authors:** Aimun Qadeer Shah, Noella Noronha, Robert Chin-See, Christina Hanna, Zeest Kadri, Amn Marwaha, Neetu Rambharack, Jeremy Y. Ng

**Affiliations:** Department of Health Research Methods, Evidence, and Impact, Faculty of Health Sciences, McMaster University, Hamilton, ON Canada

**Keywords:** complementary and alternative medicine, eHealth, integrative medicine, scoping review, telehealth, telemedicine

## Abstract

**Background:**

Telemedicine includes the delivery of health-care services and sharing of health information across distances. Past research has found that telemedicine can play a role in enhancing complementary, alternative, and integrative medicine (CAIM) while allowing the maintenance of cultural values and ancestral knowledge. This scoping review synthesized evidence regarding the use of telemedicine in the context of CAIM.

**Methods:**

Following Arksey and O’Malley’s scoping review framework, CINAHL, PsycINFO, MEDLINE, EMBASE and AMED databases were searched systematically. The CADTH website was also searched for grey literature. Eligible articles included a CAIM practice or therapy offered through telemedicine, with no restrictions placed on the type of telemedicine technology used. Inductive thematic analysis was conducted to synthesise common themes among the included studies.

**Results:**

Sixty-two articles were included in this synthesis. The following themes emerged: 1) the practitioner view of CAIM delivered through telemedicine, 2) the patient view of CAIM delivered through telemedicine, and 3) the technological impacts of telemedicine delivery of CAIM.

**Conclusions:**

Studies have shown that telemedicine delivery of CAIM is feasible, acceptable, and results in positive health outcomes. Some barriers remain such as the presence of chronic illness and morbidity, inability to form strong patient-provider relationships relative to face-to-face approaches, and technological difficulties. Future intervention research should focus on reducing such barriers, as well as explore which patient population would realize the greatest benefit from CAIM delivered via telemedicine, and the impact of interventions on providers and caregivers.

## Background

Telemedicine is used today as an umbrella term encompassing the delivery of health-care services and the exchange of health-care information across distances, with the help of a wide variety of technology [1, 2]. The word telemedicine has been supplemented by terms such as telehealth, online health, and more recently, e-Health [1]. It is widely recognized that there is no single, definitive definition of telemedicine [3]. However, the World Health Organization describes telemedicine as: “the delivery of healthcare services by healthcare professionals over a distance involving the exchange of information related to diagnosis, treatment and prevention of diseases and injuries, research and evaluation, and for continuing the education of healthcare providers, all with the goal of advancing health and the healthcare system” [4].

While technology continues to advance, infrastructure and legal barriers remain within the field of telemedicine [5]. Despite these barriers, the implementation of telemedicine measures remains promising with the potential to significantly reduce healthcare expenditures, especially in rural or remote areas such as Northern Canada where the cost of healthcare prevails [6]. Past research has found that telemedicine can also play a role in enhancing complementary, alternative, and integrative medicine (CAIM), allowing the maintenance of cultural values and ancestral knowledge [7]. However, more research is warranted to understand the use and impacts of telemedicine for CAIM.

CAIM is typically described as therapies used together (complementary), in replacement (alternative) of conventional Western medicine, or as the combining of both conventional and unconventional therapies in a coordinated way (integrative) [8, 9]. CAIMs encompass a broad range of approaches that commonly include natural products (e.g., vitamins, herbs, probiotics), mind and body practices (e.g., yoga), and traditional forms of medicine (e.g., traditional Chinese medicine) [9]. The use of telemedicine for the delivery of CAIM holds unique potential to increase access to CAIM practices for those living in remote areas or with accessibility challenges. Greater accessibility to CAIMs through telemedicine may potentially improve clinical outcomes, decrease patient healthcare utilisation, and enhance patient satisfaction with mental health and chronic disease management [10, 11]. The continued use of CAIM in treatment plans, and the growing use of telemedicine as an avenue to extend healthcare, particularly for remote and rural communities, justifies the need to investigate how telemedicine is used in the context of CAIM. Thus, the purpose of this scoping review is to understand the breadth of the literature regarding telemedicine used in the context of CAIM, to inform future areas of investigation and practice.

## Methods

This review was conducted to understand how telemedicine is used in the context of CAIM. Arksey and O’Malley’s five-stage scoping review framework [12] was utilised and also supplemented with additional scoping review guides [13–15]. The five steps were as follows: 1) identify the research question, 2) identify the relevant studies, 3) select relevant studies, 4) chart data, and 5) collate, summarise, and report the results.

### Step 1: Identify the research question

The research question for this scoping review was as follows: “How is telemedicine used in the context of CAIM?” For the purposes of this review, telemedicine was defined based on recent, well-cited review articles, as the application of any online or digital service such as Facebook live groups, Twitter, phone, mobile application, and websites, to enhance health-care management [16–18]. CAIM was defined using the operational definition provided by the Cochrane Complementary Medicine group, which included a list of therapies that were classified as complementary, alternative, or integrative medicines [19, 20]. All CAIMs discussed met the Cochrane Complementary Medicine group’s definition. In this review, all included studies contained at least one type of telemedicine being used for at least one type of CAIM.

### Step 2: Finding relevant studies

A preliminary scan of the literature indicated that academic literature on this subject area was sparse. We devised a systematic search strategy as shown in Table 1. CINAHL, PsycINFO, MEDLINE, EMBASE and AMED databases were searched on October 12, 2020. The CADTH website was used to search for grey literature, and was also searched on the same day [21]. Primary research articles were considered, and relevant reviews were used to source additional eligible primary research articles.

**Table 1:** MEDLINE search strategy for studies investigating how telemedicine is used in the context of CAIM, executed October 12, 2020

Database: OVID Medline Epub Ahead of Print, In-Process & Other Non-Indexed Citations, Ovid MEDLINE(R) Daily and Ovid MEDLINE(R) 1946 to Present
Search Strategy:
1 (alternative medicine* or alternative therap*).mp. (26,008)
2 (complementary medicine* or complementary therap*).mp. (22,731)
3 exp Complementary Therapies/ (229,053)
4 (integrat* adj1 (medicine or therap*)).mp. (4727)
5 exp Integrative Medicine/ (1589)
6 naturopath*.mp. (1679)
7 exp Naturopathy/ (1000)
8 acupunctur*.mp. (30,752)
9 exp Acupuncture Analgesia/ or exp Acupuncture Points/ or exp Acupuncture Therapy/ or exp Electroacupuncture/ or exp Acupuncture/ (25,468)
10 (chiropract* or spinal manipulation*).mp. (9278)
11 exp Chiropractic/ or exp Manipulation, Chiropractic/ (4106)
12 (herb* adj1 (medic* or therap* or supplement*)).mp. (28,602)
13 exp Medicine, East Asian Traditional/ or exp Medicine, Chinese Traditional/ or exp Herbal Medicine/ or exp Plants, Medicinal/ or exp Phytotherapy/ (109,946)
14 tcm.mp. (12,047)
15 exp Drugs, Chinese Herbal/ (43,983)
16 traditional Chinese medicine.mp. (23,455)
17 exp Medicine, Ayurvedic/ (2234)
18 ayurved*.mp. (7373)
19 acupressure.mp. (1546)
20 exp Acupressure/ (763)
21 applied kinesiolog*.mp. (102)
22 exp Kinesiology, Applied/ (312)
23 herbalism.mp. (152)
24 exp Osteopathic Medicine/ or exp Manipulation, Osteopathic/ (4055)
25 osteopath*.mp. (7792)
26 exp Mind–Body Therapies/ (50,758)
27 mind–body*.mp. (5608)
28 exp Yoga/ (2870)
29 yoga.mp. (6357)
30 or/1–29 (382,977)
31 exp Telemedicine/ (30,218)
32 exp Diagnosis, Computer-Assisted/ or exp Surgery, Computer-Assisted/ (108,122)
33 exp Electronics, Medical/ (6455)
34 exp Pharmaceutical Services, Online/ (102)
35 exp Telenursing/ (220)
36 exp Remote Consultation/ (4924)
37 (("computer-assisted" adj2 (medicine* or therapy*)) or e-consultation or e-medicine or ((electronic or internet* or mobile*) adj2 (care consult* or intervention* or medicine* or monitor*)) or mhealth or teleabortion or teleaudiology or telecardiology or telecare or teledentistry or teledermatology or telediagnosis or telehealth* or telemedic* or teleneurology or teleneuropsychology or telenurs* or telenutrition or teleophthalmology or telepathology or telepharm* or telepsychiatry or telepsychotherapy or teleradiology or telerehab* or telescreen or telesurgery or teletherap* or teletrauma*).ti. (18,292)
38 or/31–37 (151,052)
39 30 and 38 (729)
40 limit 39 to english language (628)

### Step 3: Selecting the studies

Records were included if they mentioned CAIM and telemedicine, with no restrictions placed on the type of telemedicine strategy. Records were excluded if they were 1) non-academic or non-scholarly sources (e.g., websites, blogs, news articles), 2) found outside of bibliographic database searches (e.g., unpublished theses and dissertations), or 3) conference abstracts. Only articles published in English were included. Three authors (NN, AM, CH) first pilot-screened titles and abstracts independently and in duplicate, and then met to verify the appropriateness of the inclusion criteria. Next, the three authors completed independent screening of articles for eligibility by title and abstract, and full text. Disagreements were resolved with discussion with the senior author (JYN) and in the case that consensus was not reached, eligibility was determined based on majority vote.

### Step 4: Charting the data

Articles that met inclusion criteria were critically reviewed using Arksey and O’Malley’s descriptive-analytical narrative method [12]. The following information was extracted by three authors (NN, AM, CH): title, author, year, country, study setting, study design, population type and sample size, type of CAIM used, type of telemedicine used, primary and secondary outcomes and how they were measured, main findings, challenges encountered, and conclusions. Authors then met to resolve any data discrepancies. Later, four authors (AQS, NR, RCS, ZK) reviewed the data extraction as a quality measure.

### Step 5: Collating, summarising and reporting results

Charted data was summarized in table format, and thematic and descriptive data was analysed (NN, AM, CH). A thematic analysis was conducted to present a narrative related to the research question and highlight knowledge gaps in the current literature (AQS, NR, RCS, ZK). Themes were discussed in consultation with NN and JYN, who have prior experience in conducting thematic analyses.

## Results

### Search results

**Fig. 1:**
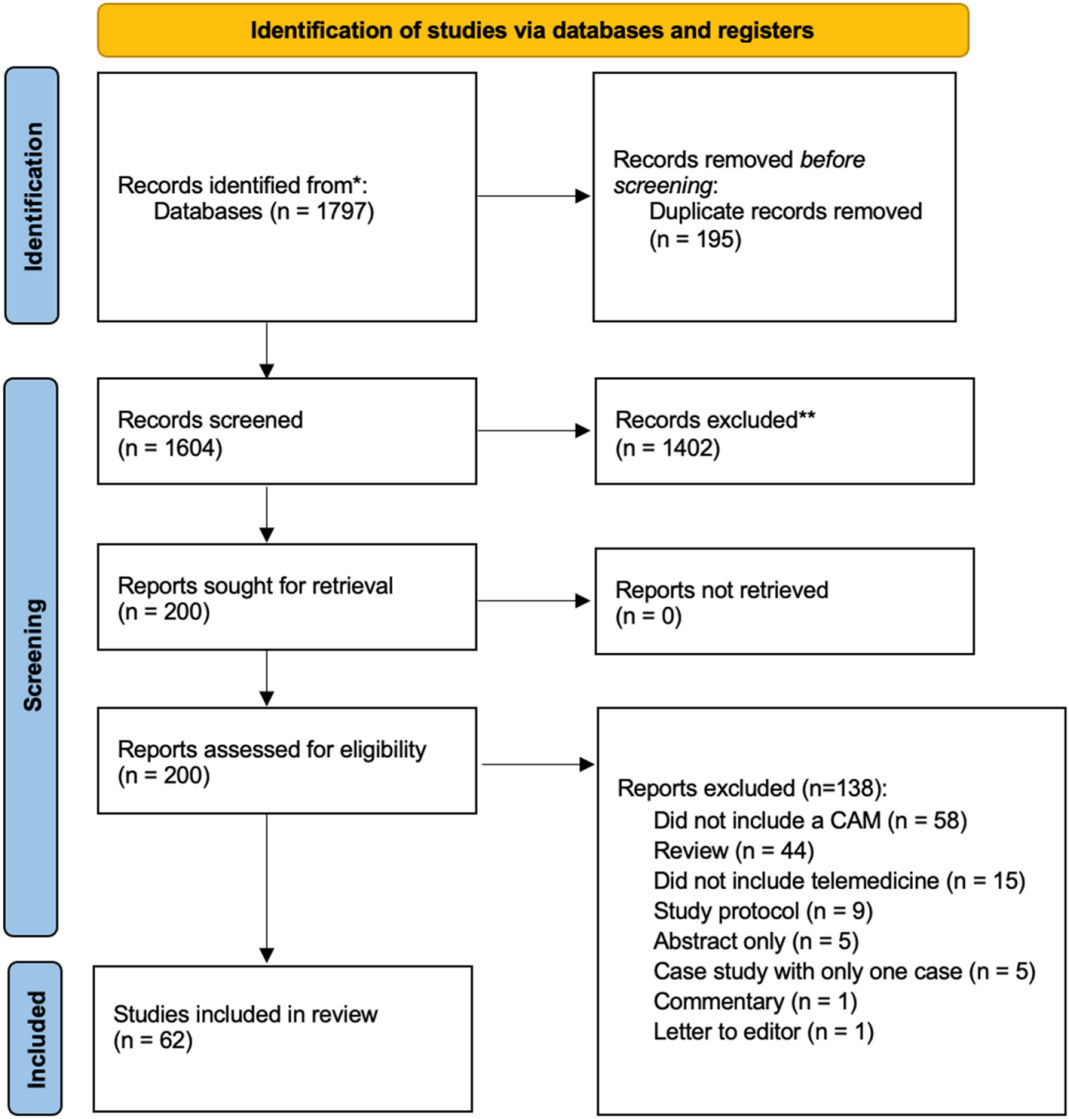
PRISMA diagram displaying the search strategy and selection process [22]

Searches identified a total of 1797 records, of which 1602 were unique. A total of 1402 titles and abstracts were eliminated, leaving 200 full-text articles to be considered. Of these, 138 were ineligible for the following reasons: did not include a CAIM (*n* = 58) or did not include telemedicine (*n* = 15), review (*n* = 44), research protocol (*n* = 9), conference abstract (*n* = 5), case study (*n* = 5), commentary (*n* = 1), or letter to editor (*n* = 1), leaving a total of 62 eligible studies which are included in this scoping review. A breakdown of study filtration through the inclusion exclusion process can be found in Fig. 1.

### Eligible article characteristics

Eligible articles were published from 1999 to 2020, and originated from the United States (*n* = 34), Italy (*n* = 4), the United Kingdom (*n* = 3), South Korea (*n* = 3), Canada (*n* = 2), China (*n* = 2), Norway (*n* = 2), Taiwan (*n* = 2), Australia (*n* = 2), France (*n* = 1), Germany (*n* = 2), Iceland (*n* = 1), Israel (*n* = 1), and Switzerland (*n* = 1). One article included participants from both the US and the UK [23], and another study included collaboration between Austria and China [24]. Of the 62 articles included, all were primary research articles focused on, development of a telemedicine technology or processes for CAIM (*n* = 11), analysis of the data collected by a telemedicine technology for CAIM (*n* = 26) and/or, analysis of usability, acceptability, or feasibility of existing telemedicine software (*n* = 25). The characteristics of all eligible articles can be found in Tables 2, 3, and 4.

**Table 2:** General characteristics of the included studies investigating the use of telemedicine in the context of CAIM (*n* = 62)

Author and Year Published	Country	Aim	Methodology	Telemedicine Type	CAIM Type	Outcome Assessment	Strengths	Challenges	Limitations	Conclusions
Addington et al., 2018 [25]	USA	To conduct a pilot trial of internet-based, cancer-adapted yoga for women receiving breast cancer treatment	Pilot trial	Videoconferencing	Yoga	Feasibility and acceptability as indicated by 1. quantitative: enrollment rate, retention, adherence, satisfaction; 2. qualitative: feedback from program evaluation forms and telephone interviews	Data from qualitative interviews can inform development of future trials	Recruitment and retention of women due to technological difficulties, scheduling conflicts, treatment related fatigue, forgetfulness due to “chemo brain.”	Small sample size, no control group	Technological and cancer-related barriers remain in internet-based oncology interventions
Armin et al., 2020 [26]	USA	To create guided imagery program material that is inclusive, including for men and racial/ethnic minority tobacco users	RCT	Telephone	Guided Imagery	In the development phase: small group and individual semi-structured interviews to understand how participants perceived the program. Pilot test: patient satisfaction on 1–5 Likert scale	Program development was informed by experts, community members, small group interviews, and focus groups. Feedback from pilot testing enabled improved program delivery	Lack of participant understanding of quit lines may be a barrier to using them	Lack of representativeness of the sample, “real time” rapid revisions limited the ability to make significant changes that may have improved outcomes	This study is the first of its kind to gather qualitative information on a telephone-delivered, guided-imagery intervention, while respecting cultural diversity
Berman et al., 2009 [27]	USA	To assess the feasibility of delivering self-care tools to older adults via the internet and to document changes in pain and ability to manage chronic pain	RCT	Website	Mind–Body Training	Pain using the BPI, self-efficacy using the PSEQ, depression using the CES-D, anxiety using the STAI-6, self-care, self-awareness to pain using the PAQ, awareness of, and satisfaction and use of the intervention	The intervention appealed to various age groups. The characteristics volunteer participants may be helpful for identifying target populations and developing outreach strategies	Study participants were more likely to be female, suggesting the intervention may be more likely to appeal to women than men	Small sample size, short length of intervention (6 weeks), improvements potentially due to social desirability effects or starting with more severe pain	Older adults with chronic pain can benefit from a short-term, online bind-body intervention
Bombardier et al., 2013 [28]	USA	To determine whether an intervention to increase physical activity might be an effective treatment for major depression in people with MS	RCT	Telephone	Physical activity	Depressive symptom using the HAM-D, SCID, SCL-20, and PANAS. Significance was considered at least a 50% reduction in the HAM-D compared to the wait-list control group	Telephone-delivered interventions can help overcome common barriers to participation. The intervention had low dropout rate, high treatment adherence, and appears safe, tolerable, and feasible	No attention control group. The wait-list control group was offered the intervention after the 12-week assessment, so it was not possible to compare outcomes between groups at 24 weeks	The appropriateness of this intervention for those with greater MS-related disability, more severe depression, or increased suicidal risk is uncertain. A subjective, self-report measure of physical activity was used	Telephone-based counseling has potential to promote physical activity in people with MS, and can help overcome some barriers to speciality care
Cavalera et al., 2019 [29]	Italy	To test the efficacy of an online MBI to improve QoL, psychological well-being, sleep, and fatigue in people with MS	RCT	Website, Skype videoconferencing	Meditation	QoL using the MSQOL-54. Secondary outcomes of anxiety and depression assessed with the HADS, fatigue assessed with the MFIS, sleep measurements obtained with MOSS scale	Adequate sample size, active control group, accessible for those with physical disabilities	Technological issues for at least one participant each session, such as slow internet connections and the interaction with the computer interface	Complete participant blindness wasn’t possible. Potential sample bias as only those with an electronic device were selected	An online MBI could be an effective psychological treatment for the promotion of well-being in those with MS, although the lack of lasting effects requires development of new strategies
Cheung et al., 2018 [30]	China	To design a Qigong app for delivery of training to the general public in Hong Kong and to examine usability and acceptance of the app	Pilot trial	Mobile app	Qigong	Usability of the app using the SUS, user acceptance measured in terms of attitude, perceived usefulness, intention to use, and satisfaction. Objective measures of usability using app navigation tasks, and noting success rate and task time	End users were involved in the early stages of design, allowing developers to identify important design challenges. Overall usability scores were within an acceptable range	Those of older ages gave lower ratings for usability and perceived ease of use, while experienced smartphone users had less intention to use the app and were less satisfied it	Convenience sample, most participants were female, and no qualitative feedback was obtained during the main test	Evidence found that usability and acceptance of a training app can enhance participants’ access and motivation to practice Qigong
Davis et al., 2015 [31]	USA	To assess the feasibility of providing mindfulness training online to smokers	Pre-post intervention	Telephone, web-based videos and audio recordings	Mindfulness training	Feasibility measures: phone call completion and length, video completion, website time, minutes of daily meditation, and mindfulness practice. Self-report measures: nicotine dependence using the FTND, mindfulness using the FFMQ, and depression using the DASS. Abstinence measures: carbon monoxide breath test at baseline, 4- and 24-weeks post-quit attempt	Provides evidence for a novel intervention for smokers. Has potential for large-scale dissemination, and may complement quit-lines well	While behavioural treatment was intensive, pharmacotherapy was relatively non-intensive, study was insufficiently powered for a few of the outcome measures	Small sample size, lack of a control group, potential selection bias as participants were required to have internet access and were selected from a pool of individuals who didn’t have time for in-person sessions	Web-based mindfulness training is feasible for smokers. Further study may be beneficial
Dimitropoulos et al., 2017 [32]	USA	To report on the feasibility of using telehealth for direct intervention in a Prader–Willi syndrome sample	Prospective cohort	Videoconferencing	Play based therapy	Parents’ acceptability of the program using the modified BIRS, and open-ended questions regarding limitations of the telehealth mode	The BIRS has strong psychometric properties. Children enjoyed the program, and parent acceptability reports were congruent with study metrics for feasibility and acceptability	Troubleshooting technological issues, difficulty finding a conducive “space” and time for intervention sessions, and the need to adjust protocol when children were noncompliant or emotionally upset	Small sample size, and telehealth may only be suitable for those at least minimally verbal, able to attend without in-person support, and do not have significant behavioural concerns. Not all parents completed the BIR survey	Findings support using telehealth in rare disorders, and delivering interventions directly to children with developmental delays
Donesky et al., 2017 [33]	USA	To determine the feasibility and clinical outcomes of an 8-week home-based yoga program, conducted via video-conferencing in a sample of patients with both COPD and HF	Controlled non-randomized trial	Videoconferencing	Yoga	Physical function defined as muscle strength and endurance, QoL using the St. George’s Respiratory Questionnaire and the KCCQ, and symptoms of depression, dyspnea, and insomnia evaluated at baseline and after study completing using the PHQ-8, the Dyspnea-12 questionnaire, and the GSDS	Participants were adherent, able to safely participate, and found classes enjoyable. Conducting assessments at home minimized missing data, and increased intervention access for this frail population	Technical issues such as with log-in, delays in connecting to the server, frozen screens, and audio or video. Some older, income-restricted adults had slow internet access, and lack of basic technical understanding	Small sample size, risk of sample bias as a convenience sample was used, reports of vital signs before and after tele-yoga sessions were not observed and there is a possibility they were fabricated to please investigators	Despite frailty, participants were able to safely perform yoga in the home setting. However, technical issues were an important hindrance to participation
Ezenwa et al., 2016 [34]	USA	To test feasibility of a guided audio-visual relaxation intervention protocol for reducing stress and pain in adults with SCD	RCT	Tablet audio-visual videos	Guided relaxation	Stress and pain using the stress intensity scale and the PAIN-ReportIt software program, respectively. Acceptability using the study acceptability scale, android acceptability scale, and an open-ended exist interview guide	Provides evidence of non-drug strategies to reduce stress, participants could use the mobile guided relaxation anywhere and anytime, and this approach can include those whose condition precludes them from travelling for research visits	Patients did not use the intervention for a total of 13 days as intended. Patients suggested the guided relaxation be customized for SCD	The mechanism through which the intervention was effective may have been via relaxation rather than stress reduction. Data was not collected from those that lost the study tablet	The study protocol appears feasible and shows promise. The results warrant a larger efficacy trial
Ferraris et al., 2020 [35]	Italy	To retrospectively describe the use of remote monitoring by e-mail during the first year of follow up on cKD in patients with GLUT1-DS and DRE	Retrospective	E-mail	Ketogenic diet	Control visits 1 month after cKD initiation, then at 3, 6, 9, and 12 months. Measurements of fasting blood ketones, compliance to the prescribed diet, and screening for potential adverse effects. All emails exchanged between the patient’s family and the keto-team during follow-up periods were analyzed	Email can be read and accessed from different sites, it enables better two-way communication between hospital specialists and other colleagues, and rapid assistance can be provided to patients	Lack of palatability of ketogenic food, qualitative and quantitative restrictions in meal preparation can be burdensome, adherence may limit participation in daily social activities for patients and families	Retrospective design, limited sample size and follow-up period, an inability to detect telephone contacts that integrate monitoring, and the lack of a control group	Constant remote e-monitoring could be a feasible and effective way for better cKD management, especially for those who live far from the treatment centre
Freeman et al., 2014 [36]	USA	To compare the effects of an intervention “envision the rhythms of life” delivered live or via telemedicine compared to waitlist control on QoL for breast cancer survivors	RCT	Videoconferencing, phone calls	Guided imagery	General health-related QoL using the SF-36 PCS and MCS scores, breast cancer-specific QoL, fatigue, perceived cognitive function, spiritual well-being, psychological distress, and sleep disturbances. Outcomes measured at baseline, 1 month, and 3 months after treatment	The program comprehensively addresses many facets of QoL. Improvements in cognitive function, fatigue, sleep disturbance, and mental health related and breast cancer-related QoL were considered clinically significant	Adherence to home practice could not be documented, limiting the ability to examine “dose effect.”	Small sample size, staffing limitations, lack of active control group, no examination of social support, making it difficult to examine or control for change in social support during study	Telemedicine is an effective and viable method to deliver a group intervention aimed at improving QOL in breast cancer survivors
Gardner-Nix et al., 2014 [37]	Canada	To investigate the effectiveness of a MBCPM program developed for a severe chronic pain population	Non-randomized control trial, original research	Videoconferencing through Ontario Telemedicine Network	Mindfulness training	Evaluated at week 1 and 10 for QoL, perceived usual pain levels, pain catastrophizing, and suffering using the SF-36, pain intensity using the numerical rating scale, the pain catastrophizing scale, and the PRISM test	N/A	High attrition rate, possibly due to participant not having to pay for the course	Absence of randomization, patients had mixed pain conditions, and self-report data	Telemedicine supports the delivery of the MBCPM program, and was effective in improving mental health and suffering
Golebowicz et al., 2015 [38]	Israel	To examine the feasibility and effectiveness of a tele-biofeedback ergonomic intervention programme among computer operators suffering from WRMSD in the workplace	Pre-post test	Remote tele-biofeedback sessions	Ergonomic biofeedback programme	Difference in pre- and post-intervention MSD scores, which included UES detected by physical examination, and pain reported in the SNQ. Bad posture risk factors were assessed by the RULA, and the DCSQ assessed psychosocial job characteristics	Unique remote communication between participants and researchers. Findings strengthen knowledge regarding the correlation between ergonomic biofeedback and reduction of pain for computer operators	The participant dropout rate was high due to poor compliance, and lack of cooperation with the examiner	Small sample size, lack of control group, not possible to differentiate between the various components of the intervention to single out telebiofeedback as an effective component	Biofeedback seems to be feasible and efficient for computer operators who suffer from WRMSD
Green et al., 2020 [39]	USA	To describe the rapid deployment of telehealth, particularly real time video conference, for chiropractic services as a response to COVID-19	Retrospective description	Videoconferencing	Chiropractic care	Described how real-time videoconferencing is operationalized, as well as the apparent effectiveness and satisfaction with the online practice	The client company and the Stanford Health Network were able to work closely together, which lowered barriers to telehealth such as insurance and company reimbursement concerns	Procedures with muscle stretch reflexes or passive range of motion cannot be performed during telehealth visits	Findings are a snapshot during the COVID-19 pandemic and the response described in this paper is likely to evolve with the expansion of telehealth and telehealth laws	Real time videoconferencing can be quickly implemented for chiropractic services, and implications for providers are described
Guétin et al., 2016 [40]	France	To assess the pain- and anxiety-reducing effects of the Music Care application in patients undergoing coronarography	Uncontrolled observational study	Mobile app	Music therapy	Before and after app use, participants rated current pain intensity and anxiety on 10-point visual analogue scales. Satisfaction was rated after the session	The music was solely recorded for this app, avoiding potential memory effects or later conditioning, distinguishing the app from other interventions	Men exhibited no to very low pain in association with the procedure, so analysis on the intervention’s pain-reducing effects in men was not able to be performed	Preintervention ratings of anxiety were quite low within participants	The smartphone based Music Care application is easy to use in reducing anxiety in patients undergoing coronarography
Hansen et al., 2015 [41]	Iceland	To determine the feasibility of using audio relaxation technique, music intervention, nature video appl with music, and nature video app without music in a clinical setting	RCT	Mobile app technology	Music therapy	Assessed state anxiety using the state version of the STAI-Form Y, pain levels using the NRS, and self-efficacy using the GSE	This novel study indicates that the Icelandic adult population is open to alternative ways of healing	There was inconsistency of nursing staff pre- and post-operatively since research assistants were not always available to work with all the participants; nurses may differ in how they administer pain medication	Small/medium sample size, control group participants did not answer baseline questions, and the free access to popular mobile technology may have lured participants to take part, creating bias	Despite non-significant findings between five groups, valuable trends towards significance and confirmed feasibility in a clinical setting were noted
Hasan et al., 2019 [42]	United Kingdom	To assess whether hypnotherapy by Skype may overcome the lack of availability of gut-focused hypnotherapy which is an effective treatment for IBS	Pre-post	Videoconferencing	Hypnotherapy	IBS severity using the IBS symptom severity score, IBS noncolonic symptom score, IBS QoL score, and HADS scores for anxiety and depression	Skype therapy increases access, as 71% of subjects claimed they would not have been able to have this form of treatment otherwise	11.8% of participants found the audio quality to be good in only half the sessions due to disturbance in network connection	Mean depression scores were not above normal range at baseline. Hypnotherapy cannot be considered as a stand-alone treatment	Skype hypnotherapy appears to be a good alternative (although slightly less effective) to face-to-face treatment in subjects who would find it difficult to otherwise access treatment
Hernandez et al., 2018 [43]	USA	To determine feasibility and acceptability of an Internet-based positive psychological intervention in hemodialysis patients with comorbid depressive symptoms	A single-arm pre-post pilot trial	Website	Mindfulness training	Feasibility using recruitment rates, refusal rates, retention rates and non-compliance /adherence rates. Acceptability using qualitative ratings of content, modality of delivery, and whether it was enjoyable, comprehensible and beneficial. Assessed depression, kidney disease QoL, and dietary adherence	Fills a critical gap in science by taking advantage of current technologies that can improve cost-effectiveness and can more easily propagate wide dissemination	Primarily text-based delivery of information may create undesirable friction or cognitive burden. This population also has a need for personal reflection exercises that don’t require extensive typing due to restrictive arm movement	Non random sampling, small sample size, and an inability to have a longer follow-up period	An innovative Internet-based positive psychological intervention represents a feasible and useful therapeutic option for hemodialysis patients with depressive symptoms
Horneber et al., 2018 [44]	Germany	To report on all telephone consultations with cancer patients or their relatives held between 1999 to 2011 along with the results from a nested feedback survey	Retrospective analysis	Telephone	CAM consultation service	Assessed substance of telephone consultations including reasons for interest in CAM consultations, the topics discussed in calls, and satisfaction with the service	Participants particular perceived the service as helpful because researchers did not hesitate to highlight risks of CAM, or where their benefits were uncertain	Relied on consultants’ documentation, which are inherently influenced by different communication styles and prioritizing of issues. Consulting about CAM cannot be separated from consulting about conventional care	Only one-third of callers completed the feedback survey, potential for sampling bias as people with higher levels of engagement in their own health or care of another person were more likely to ask for a consultation	Consulting about CAM addresses important unmet needs from cancer patients and their relatives
Houweling et al., 2015 [45]	Switzerland	To compare differences in outcomes, in spinal, hip, and shoulder pain patients who initiated care with MDs vs DCs	Retrospective double cohort	Videoconferencing	Chiropractic manipulation	Pain, patient’s global impression of change, satisfaction, and use of health-care services. Information on health care costs was extracted in a subsample from the database of an insurance provider	The first study in Switzerland to compare health outcomes and cost for patients consulting differing first-contact care providers	Although differences in pain relief scores were significant, they are likely not of clinical significance	Low response rate, lack of standardized validated outcome measures, limited or missing information on clinical/demographic characteristics	Spinal, hip, and shoulder pain patients had clinically similar pain relief, greater satisfaction levels, and lower overall cost if they initiated care with DCs, compared to MDs
Hu et al., 2013 [46]	Taiwan	To develop a cloud system to integrate EMRs and encourage communication between medical workers, and improve the quality of traditional Chinese medicine offered to hospitalized patients in medical centres	Technology development	Cloud system	Traditional Chinese medicine	Effectiveness of the system evaluated qualitatively and quantitatively by time spent preparing for acupuncture and time needed for keeping acupuncture records, punctuality of removing needles, time-saving in data integration after treatment, percentage of unremoved needles, accuracy of patient identification, human resource management, and impact on access of medical information	There is great improvement in operating efficiency shown by quantitative measures, but qualitative measures also indicate the superiority of the cloud system. Data synchronization is automatic	For wide application of the cloud system, Wi-Fi penetration rate needs to be raised, which poses a problem for many hospitals in Taiwan	The cloud system is limited to android operating systems, and servers used must be reliable and trustworthy	The contribution made by the cloud system to the traditional Chinese medicine service is multi-dimensional: cost-effective, environment-protective, performance-enhancing and more
Huberty et al., 2017 [47]	USA	To report the satisfaction and perceptions of an online yoga intervention in women who have experienced a stillbirth	Post intervention, cross-sectional and semi-structured interviews	Videos	Yoga	Experiences, perceptions, and satisfaction through surveys and semi-structured interviews	Was the first study to explore a home-based modality for management mental health in women after a stillbirth. The strategy can be largely disseminated with little resources	Barriers to participating included technical issues, lack of instructor feedback, and the lack of autonomy with the delivery approach	The number of non-completers was the same as completers (n = 26)	Online-streamed yoga may be a useful approach to deliver yoga to women who have experienced a stillbirth
Hucker et al., 2014 [48]	Australia	To evaluate an online treatment for female sexual difficulties as it relates to relationship functioning	Pre-post, cross-over design, original research	Online videos and chat groups	Mindfulness training	Sexual dysfunction or distress using the sexual function scale, personal assessment of intimacy in relationships scale, female sexual function index, and female sexual distress scale	Cross-over design, and treatment gains were maintained at 3-month follow-up	The treatment group lost 44% of participants. Relationship satisfaction scales did not address aspects such as division of labour, and financial conflict which may explain non-significant differences	Small sample sizes, potential sample bias due to the use of volunteers, and data was self-reported	The intervention resulted in significant improvement in sexual intimacy and communication, and in emotional intimacy for study group 1. Most improvement were maintained at follow-up
Kahn et al., 2016 [49]	USA	To evaluate effects of a web-based, self-directed program of instruction in mind–body-based wellness skills for veterans and their relationship partners on mental health and wellness outcomes	4-arm RCT, original research	Videos	Mindfulness and contemplative practice training	A survey package was administered at baseline, 8 weeks, and 16 weeks, including PSS, BDI, PCL-C, SCS, RSES, MSPSS, PSQI, and RDAS scales. Also assessed use of the intervention	Both veterans and partners use of the program surpassed hypothesized time of use, and there was a benefit for both men and women veterans and their partners	The instructional program did not include video closed captioning or verbatim transcripts of the audio instruction, and thus could not accommodate users with hearing limitations	The impact on clinically defined populations remains to be assessed, the follow-up period was limited to 16 weeks, and participants were required to attend an in-person launch meeting which could have caused exclusion of potential applicants	Both veterans and partners were able to learn and make sustained use of a range of wellness practices taught in the Mission Reconnect program for this population
Kemper et al., 2017 [50]	USA	To assess the dose–response relationship between the number of hours of online mind–body skills training for health professionals and relevant outcomes a year later	Natural experiment, original research	Online educational program – video based learning	Mind–body training	“Dose” of the intervention, type, number, frequency and length of mind–body practice, stress, burnout, absenteeism, mindfulness, resilience, and compassion	Findings demonstrate that the impact mind–body skills training on outcomes appears stable at least 12 months after training, and “dose” evaluation and training type may yield clinically relevant information	No data collected at baseline due to the fact that the study was conducted as an educational evaluation, and there may be differences in benefits for novice compared to experienced meditators	The study was conducted at one academic institution, there may be self-selection bias, and self-report measures were used	Mind–body skills training affect self-reported personal and professional behavior for at least 1 year after training. Increasing doses of training are associated decreased levels of negative outcomes
Kim et al., 2020 [51]	Korea	To introduce the Korean Medicine telemedicine center as a treatment option for COVID-19 patients	Retrospective review, original research	Telemedicine centre	Herbal medicine and mindfulness meditation	Collected characteristics of participants using electric medical charts, such as demographics, residence, number of treatments conducted, treatment periods and prescription periods	This is the first report in Korea to record changes in residence transition status of patients	N/A	Retrospective descriptive analysis and does not reflect information on the improvement of symptoms of patients	Telemedicine operation in response to infectious diseases in Korea is considered meaningful for efficient use of medical resources, patient management and preventing infection spread
Krampe et al., 2016 [52]	USA	To evaluate a Fuze video conferencing software connecting nursing students with older adults during a therapeutic dance-based activity	Experimental, original research	Videoconferencing	Dance therapy	Feasibility including visual and audio quality, engagement between older adults, and nursing students’ overall satisfaction. Also assessed engagement, and overall satisfaction	The group was intentionally kept small to assess initial feasibility and identify areas for improvement. Engagement lessons were learned	The audio and video components had delays, or was sometimes blurry. Some older adults were overwhelmed having to watch the screen and dance leader at the same time	N/A	Fuze is a feasible, engaging, and satisfying approach for dance-based therapy, with better audio and visual performance than Skype
Krampe, & Musterman, 2013 [53]	USA	To report a process used to introduce nursing students to a group of older adults using video call technology during a dance-based therapy session	Experimental, original research	Videoconferencing	Dance therapy	Watched participants engage and interact and gather feedback after the sessions	There was great enthusiasm from the nursing students. This study has implications for community and long-term care clinical settings, and can be replicated with minimal resources	A larger screen would be more beneficial, and it was difficult to hear the nurse and instructor over the music	Small sample size	Dance-based therapy can engage nursing students with older adults, and skype is an innovative option for patient care
Krout et al., 2010 [54]	Australia	To engage music therapy university students in collaborative song writing utilizing Skype software	Nonrandomized cross-over design, original research	Videoconferencing	Music therapy	Conducted interviews, and collected student written reflections on their experiences, such as perceived contributions the activity had to their learning	Teleconferencing via Skype fostered song writing experiences as opposed to hindering them, and the experience was not much different than collaborating face to face	Poor resolution inhibited communication through misjudged facial expressions, and there were some auditory delays	N/A	Online song-writing may offer creative solutions for facilitating song-writing between persons who are not able to do so in person and face to face
Kubo et al., 2019 [55]	USA	To assess feasibility and preliminary efficacy of a mobile/online-based mindfulness intervention for cancer patients and their caregivers to reduce distress and improve QoL	RCT, original research	Website or mobile app	Mindfulness training	Retention and adherence, and participant-reported data on distress, anxiety, depression, pain, QoL, sleep, fatigue, mindfulness, and posttraumatic growth before and immediately after the intervention. A post-intervention qualitative interview was conducted	The program does not require hiring of teachers, or a secure physical location for classes, making it widely scalable and cost-effective	The study was not powered to examine dose–effect of meditation, and it is possible that patients did not experience significant changes in mindfulness due to high baseline scores	The rate of mobile device and internet use may be higher in this population compared to the general population, no active control group, and only one person was coding transcripts which may risk bias of the results	Provides preliminary evidence regarding the feasibility of a commercially available self-paced mindfulness program for cancer patients undergoing or who have recently completed chemotherapy
Kwon et al., 2020 [56]	Korea	To introduce a Korean Medicine doctor’s pilot mental health instruction manual in telemedicine for COVID-19	Health-care Manual, original research	Videos	Mind–body medicine	N/A	N/A	Youtube videos have one-way characteristics, but mind–body modalities are more effective in interactive communication environments	Since the creation and implementation of the manual was conducted during a pandemic, it was insufficient to consider it as a proper outcome indicator	Mindfulness is a promising intervention that may be combined with telemedicine. The telemedicine manual can provide insights into intervention development
Lee et al., 2020 [57]	Korea	To determine whether short-term effects from a previous 8-week online mind body training study persist up to a month after the end of the intervention	Non-randomized controlled study, original research	Videos	Mind–body training	Occupational stress using the KOSS, stress response, emotional intelligence, the Korean version of the CDRS, the Korean version of the coping strategy indicator, and Korean version of the PANAS, and the Korean version of the state-trait anger expression inventory at baseline, 8 weeks (end of training), and 12 weeks (1 month post-intervention)	Results show that the effects on stress response, resilience, and the use of an adaptive coping strategy lasted for a month after the end of the program	It is possible that cognitive measures are less influenced by meditation than emotional measures	Volunteer recruitment risks sampling bias, and findings are restricted to female subjects	Findings provide evidence for the long-lasting beneficial effects of an 8-week mind–body training course. An online format can provide a cost-effective solutions for employees at worksites
Lester et al., 2020 [58]	USA	To examine the feasibility, acceptability, preliminary effect, and durability of a mind–body videoconferencing program for youth with neurofibromatosis against an experimental educational control	Single-blind pilot RCT, original research	Videoconferencing	Mind–body training	Feasibility and satisfaction assessed post-intervention. Physical health and psychological QoL, social relationship QoL, depressive symptoms, anxiety symptoms, and the NRS for pain assessed post-treatment and at 6-months follow-up	The sample was graphically diverse, attrition was low, and satisfaction was similar between youth with neurofibromatosis and educational controls	N/A	Small sample size, wide confidence intervals, and possibility that participants may have guessed the treatment condition despite being masked	The intervention was well accepted, highly feasible, and resulted in sustained improvement in QoL, demonstrating adolescents are receptive to and benefit from learning resiliency skills in groups via live video
Mussman, 2016 [59]	USA	To investigate the feasibility of utilizing a four-week online ehealth yoga video series to provide adults with a potential mechanism for stress management	Mixed method, original research	Videos	Yoga	Dose of exposure, perceived stress, satisfaction, intention to continuing practice of yoga, whether it was recommended to others, and qualitative findings for feasibility and acceptability	The feasibility provides evidence that EHealth yoga interventions are worthy of further investigation and provides insight on what can be improved	Completers experienced barriers such as lack of time	Small sample size, study attrition, and use of a convenience sample	Findings support the feasibility of providing online yoga e-health via four weeks of instructions
Ondersma et al., 2019 [60]	USA	To evaluate the feasibility and acceptability of two high-reach technology-based interventions: electronic screening and brief intervention and tailored text messaging, delivered alone or in combination	Exploratory feasibility trial, original research	Text messaging	Cannabis	Patient satisfaction, retention, frequency of cannabis use, and whether they were more likely to quit due to the intervention	All participants fully completed the intervention during their clinic visit, and had high ratings for ease of use, helpfulness, and likely interest to other pregnant women	Acceptability ratings were inconsistent, and it is unclear whether this can be improved with changes to the text content itself, or whether it is a function of passive text messaging being less engaging	Small sample size, and the exploratory nature of the design. Participant self-selection of texting frequency limits comparability of retention	These two high-reach intervention elements showed strong feasibility and modest to high acceptability. Future efforts evaluating efficacy are warranted
Papadaki et al., 2016 [61]	United Kingdom	To explore employees' perceptions of ability to follow the MedDiet, preferences for setting goals if asked to follow the diet, and expectations of an Internet-based, workplace MedDiet intervention	Semi-structured focus groups, original research	Website or mobile app	Mediterranean diet	A semi-structured focus group explored participants’ perceptions on perceived ability to follow the diet, goal-setting preferences, receiving feedback on goal attainment, and expectations of the website promoting the MedDiet	Deductive thematic analysis of focus group transcripts was done by three independent trained researchers	There is a need for a tailored approach to setting specific goals. Some consumed foods such as legumes, olive oil, fish, and red meat are challenging to change	Small sample size, inclusion of self-selected healthy employees with internet access, and high adherence sample which may not represent views of those whom this intervention should ideally target	An Internet-based, workplace MedDiet intervention should address adherence barriers, utilize a tailored and activate social supports. Findings provide insights for promoting the MedDiet in non-Mediterranean regions
Petersen et al., 2017 [62]	USA	To evaluate the impact of an online spiritual care educational program on pediatric nurses' attitudes toward and knowledge of spiritual care and their competence to provide it to children with cancer at the end of life	Prospective, longitudinal, original research	Website	Spiritual care	Spiritual care competence scale, the spirituality and the spiritual care rating scale	Findings demonstrate potential to improve spiritual care and spiritual care competence in pediatric oncology nurses	N/A	Use of a convenience sample, lack of a control group, potential for self-report bias and self-selection bias, the risk of testing effect with repeated administration of the same instruments	Online spiritual care educational programs may exert a lasting impact on nurses’ attitudes toward and knowledge of spiritual care and their competence to provide spiritual care to children with cancer at the end of life
Reilly-Spong et al., 2015 [63]	USA	To describe the design, rationale and feasibility results of Journeys to Wellness, a clinical trial of mindfulness training delivered in a novel workshop and teleconference format	RCT, original research	Teleconferences	Mindfulness training	Health and attitudes outcomes questionnaire at baseline, 8 weeks (end of intervention), and at 6-months follow-up, which included outcome measures of anxiety, depression, insomnia, health-related QoL, mindfulness, worry, fatigue, and kidney disease QoL. Feasibility and acceptability were also assessed	High attendance and completion strengthen planned comparisons of the telephone-adapted intervention. The interventions were delivered as planned without the need for ad hoc revisions	N/A	Sessions were not audio-recorded for later verification of outcomes. Participants engagement was rated by the leader of the intervention, not an independent rater	Teleconference mindfulness based stress reduction is feasible, and may be useful to people with a wide spectrum of health conditions
Rickhi et al., 2015 [64]	Canada	To evaluate the effectiveness of an 8-week online spirituality informed e-mental health intervention on depression, spiritual well-being, and self-concept, in adolescents/young adults with mild to moderate MDD	RCT, original research	Video modules	Spiritual care	Depression severity, spiritual well-being, and self-concept measures assessed through semi-structured interviews were measured at baseline, 8 weeks, 16 weeks, and 24 weeks. Program completion was assessed post-intervention	The program is effective in reducing depression severity in both younger and older adults, and this is clinically significant and maintained at follow-up	Personal perceptions of spirituality resulted in recruitment challenges, and parents of potential participants questioned whether the program would challenge existing beliefs or religious values	Waitlist control group may result in overestimation of the intervention effects, participants were not blinded, the sample size was reduced due to the need to create two sub-group age samples	The program is an effective, online intervention for youth ages 13–24 with mild to moderate MDD with various life situations and in a limited way on spiritual well-being and self-concept
Rogante et al., 2010 [65]	Italy	To investigate the therapist’s POV on the use of surface electromyography with biofeedback for telerehabilitation, and general acceptability for the patient and therapist	Case study, original research	Surface electromyograph with audio biofeedback	Physical rehabilitation	Functioning based on the Action Research Arm test (ARA), which has a range 0–57	Can allow the patient to train at any time, more patients can be treated at the same time, and patients considered the treatment highly personalized	The system showed poor software and hardware usability, mainly because it often required on-site therapist intervention, and patient tasks were too complex for practical use	A larger study with people in various stages of stroke disease is needed to evaluate clinical effectiveness	There were some differences between the patient and the therapist about the ease of use of the equipment, but there was general agreement on the usefulness of the system, and overall opinion
Rosmarin et al., 2010 [66]	USA	To evaluate the efficacy of a spiritually integrated treatment for subclinical anxiety in the Jewish community	RCT, original research	Website	Spiritual care	General religiousness and life change at pre-treatment. Stress, worry, depression, intolerance of uncertainty, spiritual outcomes, Jewish religious coping, and perceptions of treatment were measured pre- and post-treatment. Satisfaction was assessed post-treatment	First study to investigate efficacy of a spiritual integrated treatment in the Jewish community, and in electronic format. Effect sizes were large for primary and secondary outcomes except for spiritual outcomes	N/A	Reliance on self-report measures of symptoms, generalizability to clinical populations and to face-to-face implementation is not known	Results offer initial support for the efficacy of spiritual integrated treatment for subclinical anxiety symptoms among religious Jews
Rybarczyk et al., 1999 [67]	USA	To compare two mind–body wellness interventions for older adults with chronic illness: classroom versus home instruction	RCT, original research	Videos	Mind–body medicine	Medical symptoms checklist, frequency of sleep difficulties, short-form McGill Pain questionnaire, anxiety, depression, health locus of control scale, health-promoting lifestyle profile, life satisfaction, and satisfaction with the total care they received	A reduction in the number of patients who met the criteria for significant levels of anxiety provide support of clinically meaningful findings. The program had lower cost and greater accessibility	N/A	Did not assess the long-term benefits, potential sample bias due to high levels of attrition (60%) during the recruitment process, and a lack of an active control group	A lower cost, more accessible home study version of a mind–body wellness program can be an effective alternative to classroom instruction
Sarah et al., 2019 [68]	Germany	To investigate the adherence to yoga as an antihypertensive intervention through telerehabilitation	RCT, original research	Telephone	Rehabilitation and yoga	Yoga adherence at six and twelve months, blood-pressure, endothelial function and heart rate, and health-related QoL	Adherence can be doubled by a simple telephone based intervention following inpatient rehabilitation	N/A	Only male patients were used, generalizability is limited as patients were recruited from one inpatient rehabilitation center	For this middle-aged male low-education cohort, a telephone program to enhance yoga practice at home is feasible and effective in supporting long-term adherence as a non-pharmacologic intervention
Seidler et al., 2017 [69]	USA	To investigate feasibility of a telerehabilitation approach to group tango instruction for people with PD, and compare key outcomes from a class taught virtually to an in-person	Controlled, prospective study, original research	Two-way live video and audio conferencing	Dance therapy	Feasibility using participant retention, adherence, and adverse events. Secondary outcomes measured balance, motor sign severity, and gait scored by trained, blinded raters	First study to investigate a telerehabilitation approach to group adapted tango instruction for individuals with PD. The intervention can address barriers to access for individuals living outside of major metropolitan areas	Remote instructors have potentially limited ability to recognize and address safety issues	Small sample size with mild to moderate PD which limits generalizability to people with severe deficits, and exclusion of those with overt dementia limits conclusions for more cognitively involved patients	A telerehabilitation approach to group adapted tango instruction was feasible and produced similar improvements to in-person instruction on measures of balance and motor sign severity in people with PD
Selman et al., 2015 [23]	USA, United Kingdom	To inform intervention refinement for future studies of Tele-interventions in advanced disease populations	Non-randomized control trial, original research	Videoconferencing	Yoga	Semi-structured qualitative interviews between 1 week and 3 months post-treatment, covering symptoms, function, motivations, expectations of participating, views and experiences of yoga, views of the battery of outcome measures, and suggestions for improvement. Assessed dementia, cardiomyopathy, depression, sleep, and interoceptive awareness	Adds to the evidence about designing and evaluating complex interventions based on complementary therapies and seriously ill populations	Some participants had difficulties due to back problems, posture issues, getting off the floor, and preference for a chair-based class. Having to rearrange furniture was inconvenient. Some connectivity issues arose	A convenience sample was used, small sample size, and the control group was not matched in terms of time commitment	Tele-Yoga is an acceptable and appropriate intervention in participants with HF and COPD and further research is warranted to refine the technology used in its delivery
Shrier et al., 2014 [70]	USA	To gather input from youth and providers on how youth who use marijuana frequently may experience frequent mobile self monitoring and responsive messaging	Qualitative, semi-structured interviews, original research	Mobile app	Cannabis	Thematic analysis was used to examine youth and provider perspectives on the mobile intervention. Participants were asked about views on the experience of using a mobile device to answer questions about themselves, and whether they thought youth would find the messages annoying or too repetitive	By repeatedly promoting use of alternative strategies to manage triggers, the messages would encourage real-time practice of new skills and behaviours in response to actual experiences	Both youth and providers thought it would be important to develop more individualized, personal messages	Small sample size from a single geographic region, convenience sample, potential sample bias as participants were willing to inform research on a mobile-device based interventions	Results suggest that mobile technology is a promising tool for brief interventions to reduce youth cannabis use and warrants further development
Simpson et al., 2002 [71]	United Kingdom	To evaluate whether hypnosis could be successfully applied via videoconferencing, with a view to incorporating it in future treatment programmes for patients with mental health problems in remote areas	Pilot study, original research	Videoconferencing	Hypnosis	Feedback was elicited in short interview and qualitative questionnaire format after the session	Patients rated the screen image and sound quality as high. 4 patients expressed a preference for video-hypnosis over face-to-face sessions (3 had no preference)	Factors that interfered with experience included brightness of fluorescent lighting in the room, external noises such as strong winds and banging doors, and the room not being sufficiently warm	Larger, controlled trials will be required to verify results	Provides preliminary results to support the effectiveness and acceptability of video-hypnosis for those living in remote areas
Singh et al., 2017 [72]	USA	To assess the feasibility of using Tele-health technology to train teachers in a rural school district on mindfulness-based procedure, measure the fidelity of the teachers teaching the procedure, and assess effectiveness of the students’ use of the procedure	Pre-post, longitudinal, original research	Social media platforms (WhatsApp and Google Hangouts)	Mindfulness training	Teacher aides collected data using an iPhone app, on verbal and physical aggression acts. Inter-rater agreement was defined as both teacher aides recording the same verbal and physical aggressive acts at about the same time	All 3 teachers were able to successfully learn and practice using the procedure for at least a month before teaching it to students. The procedure is low intensity, and does not require extensive training or expert supervision	Mixed-method data is needed to intensively study feasibility, acceptability, and effectiveness of the intervention	Small sample size	Tele-health may be effective to providing training and therapy to caregivers in remote locations that cannot readily access specialists. The program can be taught to teachers, who can teach their students to successfully use it as a self-management strategy
Stubberud et al., 2020 [73]	Norway	To develop and investigate the usability of a biofeedback treatment smartphone app for adolescent migraine sufferers	Prospective, open-label development and usability study, original research	Mobile app	Biofeedback	Assessed average number of hours of daily smartphone use, general experience with apps, experience with wearable sensors, usability, and physiological measures	The optimizing algorithm makes it better than traditional monitoring, the intervention was developed by multidisciplinary experts, and the target group was involved during the development process	Biofeedback requires several rounds of exposure to master	The first 2 usability cycles were conducted in a controlled environment, which was not fully representative of its intended use, moderate sample size, attrition	An app for young migraine sufferers to receive therapist-independent biofeedback underwent a rigorous development process and usability and feasibility testing. It is now ready for clinical trials
Tan et al., 2013 [74]	USA	To evaluate the feasibility and effects of an innovative treatment for women veterans residing in rural settings suffering from chronic pain and/or depression associated with trauma	Pre-post, original research	Video-teleconferencing	Biofeedback training	PTSD using the PCL-C, depression, pain intensity and unpleasantness, and sleep disturbances. Also reviewed patient logs detailing date and duration of practice, and pre-post practice ratings of pain intensity and unpleasantness	No technical problems encountered, and the quality of the communication was good. Patient focus groups indicate that treatment via videoconferencing was just as effective as if it had been in-person	Woman veterans presented with multiple symptoms and issues, making it a challenging group to investigate	Small sample size, and the study was not designed to identify factors that predict the use of the “stress eraser” device	It is feasible to provide treatment to women veterans in rural areas using video-teleconferencing technology between larger VA medical centers and facilities at CBOCs in rural settings
Thompson et al., 2015 [75]	USA	To assess the effectiveness of Project UPLIFT for reducing depressive symptoms and preventing the incidence of depressive episodes in adults with epilepsy; and to expand use to three additional states	RCT, cross-over design, original research	Web or telephone	Mindfulness training	Depressive symptoms using four different measures, knowledge and skills for depression, depression coping self-efficacy, self-compassion, the satisfaction with life scale, and QoL	This is one of the first studies demonstrating effectiveness of a preventive intervention for depression that is distance delivered	N/A	Limited power for detecting smaller differences in self-efficacy and self compassion or changes at follow-up, no active control	Distance delivery of group MBCT can prevent episodes of MDD, reduce Depression symptoms and increase life satisfaction in people with epilepsy. This intervention is modifiable for persons with other chronic diseases and other disparity populations
Tkatch et al., 2017 [76]	USA	To test the feasibility of an online mindfulness meditation intervention for community-dwelling older adult caregivers and to evaluate its impact on QoL, caregiver burden, and psychological well-being	Feasibility trial, pre-post study, original research	Phone and web-interface to an online learning platform	Mindfulness training	Caregiver burden using the Zarit Short Burden Interview, QoL, and psychological well-being including stress, anxiety, loneliness, and social support	The inclusion of additional psychological variables added a mechanism for the impact of reduced caregiver burden and mental/pscyhological well-being, improves access for those with transportation limitations, and is relatively low cost	The intervention may not be ideal for all older caregivers because some individuals may prefer an opportunity to utilize resources outside their homes	Small sample size, lack of control group, and potential sampling bias as participants were recruited from an existing caregiver support group	The intervention reduced caregiver burden, perceived stress, anxiety, and loneliness and improved mental well-being. Online interventions offer flexibility for caregivers regardless of their responsibilities
Tucker et al., 2008 [77]	USA	To determine the effects of telephone-based coaching and a weight-loss supplement on the weight and body fat of overweight adults	RCT, original research	Telephone	Exercise coaching and vitamin B-based weight loss supplement	Body fat in grams measured by dual energy x-ray absorptiometry, and body weight measured at three time periods. Assessed compliance post-intervention	Large sample size, random assignment, low dropout rate, use of a placebo and double-blind strategy, and measurement of body fat changes using dual energy x-ray absorptiometry	N/A	Little ethnic diversity, and only a 4-month study duration	Both coaching and supplement treatments, separately and in combination, helped subjects lose weight & body fat. Adults can be motivated to change behaviours over the phone
Uebelacker et al., 2018 [78]	USA	To test the feasibility and acceptability of an online yoga intervention for individuals with mood disorders	Experimental study, original research	Video	Yoga	PANAS for positive and negative affect immediately after viewing the yoga video, feedback questionnaire assessing overall feasibility and acceptability, adherence, dislike vs like, how likely they would be to participate in future online yoga programs, and qualitative feedback	N/A	Attrition possibly due to boredom or disinterest, or lack of sufficient understanding of the materials. Some participants found the positions were slightly difficult, or had technical issues	Only one yoga class provided, relied on self-report measures of adherence, may be too physically challenging for those with physical limitations or that have a difficult time moving	Offers preliminary support that online yoga is well-tolerated, acceptable, and associated with decreased negative affect in a subset of individuals with mood disorders
Vederhus et al., 2020 [79]	Norway	To examine whether a Norwegian Cannabis Cessation app reaches a broader or different user group compared to community-based Cannabis Cessation programs	Cross-sectional, original research	Mobile app	Cannabis	Severity of dependence for cannabis, mental distress using two versions of the Hopkins Symptom Checklist, days of cannabis use, self-efficacy of quitting, and general well-being	Findings suggest that the app reached some people who were not as likely to attend formal services, and who would possibly find it more problematic to seek face-to-face services (i.e. women)	Some app users might have needed more support than an app can offer. The majority of cannabis smokers also used nicotine, implying a need to address nicotine use in treatment. More interactivity might be needed on the app	Cannot interpret causality in a cross-sectional design, and those with sub-threshold cannabis use disorder were excluded from analyses even if they perceived their condition as problematic	The app can be an alternative for those who are not yet prepared to seek treatment in formal healthcare services. The app was able to capture an expanded segment of the cannabis-using population
Vranceanu et al., 2016 [80]	USA	To test the feasibility, acceptability, efficacy, and durability of a mind–body program for neurofibromatosis vs an attention placebo control for neurofibromatosis, both delivered via group videoconferencing	Single-blind RCT, original research	Videoconferencing	Mind–body medicine	Primary outcomes: physical health, psychological QoL. Secondary outcomes: social relationships QoL, environment QoL, depression, anxiety, pain intensity, and pain interference. Assessments occurred at baseline, posttreatment, and at 6 months follow-up	Included a geographically diverse sample of patients with Type 1 and Type 2 neurofibromatosis, and schwannomatosis, no attrition in the treatment group, and interest in participation and feasibility was superior compared to an in-person pilot trial	N/A	Moderate or small effect sizes went undetected, only half of participants reported moderate to severe pain at baseline, resulting in higher powered analysis for this subsample, only one therapist provided treatment	The intervention delivered via videoconferencing was highly feasible and accepted by patients, and resulted in sustained improvement in QoL
Wang et al., 2011 [24]	Austria, China	To examine the use of tele-acupuncture for quantifying the effects of heart rate variability in poststroke rehabilitation	Pilot study, original research	Internet, software	Acupuncture	Mean heart rate, total heart rate variability, and low frequency/high frequency heart rate variability ratio	Provides evidence that this new methodological procedure of tele-acupuncture has positive effects on heart rate variability and therewith on the state of health	N/A	This is not a randomized clinical trial, and does not use a personalized acupuncture scheme	Based on heart rate variability analysis, tele-acupuncture between China/Harbin and Austria/Graz over a distance of about 8,500 km is no longer a future vision; it has become reality
Wang et al., 2016 [81]	China	To examine the effects of music intervention on sleep quality in community-dwelling elderly people	RCT, original research	Music database on MP3 player, telephone follow-ups	Music therapy	Sleep quality using the PSQI-C assessed at baseline, 1 month, 2 months, and 3 months after study entry	This is the first study that used music among Chinese community-dwelling elderly with poor sleep quality, and contributes new evidence	N/A	The dose of the intervention may vary as use was self-conducted, sleep quality was unable to be measured objectively, and the follow-up period is only 3 months	Music is a safe and effective nonpharmacologic approach for improving the sleep quality among community-dwelling elderly, especially for sleep latency, sleep efficiency, and daytime dysfunction
Yeh et al., 2013 [82]	Taiwan	To investigate the effects of auricular acupressure alone or combined with an interactive Internet-based intervention for the management of menstrual pain and self-care of adolescents with primary dysmenorrhea	Non-randomized control trial, pre-post test, original research	Website	Auricular acupressure	Quality and intensity of pain using the SF-MPQ, and the mental distress questionnaire to assess severity of physiological symptoms	Results show that auricular acupressure combined with interactive Internet instruction is better than auricular acupuncture alone in improving self-care for pri-mary dysmenorrhea	N/A	Non-randomized study has inherent limitations, no control group, the sample was taken from one high school, and long-term effects remain unknown	Auricular acupressure alone or in combination with interactive internet instruction reduced menstrual pain and distress for primary dysmenorrhea. The interactive internet instruction group is better than auricular acupuncture alone for self-care behaviours
Zini et al., 2018 [83]	Italy	To set up an ICT intervention meant to support refractory epilepsy in patients undergoing ketogenic diet treatment at the centre	Observational, original research	Mobile app	Ketogenic diet	N/A	N/A	Intervention still does not address ketogenic diet. Functionality and a decision support system would be beneficial, and would require further testing	N/A	The health application for training patients in managing ketogenic diet also acts as a bridge connecting patients with the health care staff for coaching and monitoring purposes
Zwart et al., 2000 [84]	USA	To determine whether people receiving lay pastoral telecare would report greater positive change in spiritual well-being and church satisfaction than the control group	RCT, original research	Telephone	Spiritual care	Spiritual well-being scale administered over the telephone, church satisfaction questionnaire	Many strong relationships were developed between callers and callees	Optimal length and frequencies of telephone calls is still unknown; some participants preferred weekly calls while others requested calls once a month or every other week	Telephone administration of the spiritual wellbeing scale has not been tested for reliability or validity. Participant bias is possible when answering post questionnaire	Lay Pastoral Telecare intervention is an effective mode of providing spiritual support of an interpersonal nature to church attenders

Abbreviations: *BIRS* Behavioural Intervention Rating Scale, *BDI* Beck’s Depression Index, *BPI* Brief Pain Inventory, *CAIM* Contemporary, Alternative and Integrative Medicine, *CBOCs* community-based outpatient clinics, *CDRS* Connor-Davidson Resilience Scale, *CES-D* Center for Epidemiologic Studies Short Depression Scale, *cKD* classic ketogenic diet, *COPD* Chronic Obstructive Pulmonary Disease, *DASS* Depression Anxiety Stress Scales, *DCs* Doctors of chiropractic, *DCSQ* The Swedish Demand-Control-Support Questionnaire, *DRE* Drug resistant epilepsy, *EMRs* Electronic medical records, *FFMQ* Five-Facet Mindfulness Questionnaire, *FTND* Fagerstrom Test for Nicotine Dependence, *GLUT1-DS* glucose transporter type 1 deficiency syndrome, *GSDS* General Sleep Disturbance Scale, *GSE* General Self-Efficacy Scale, *HADS* Hospital Anxiety and Depression scale, *HAM-D* Hamilton Depression Rating Scale, *HF* Heart Failure, *IBS* Irritable Bowel Syndrome, *ICT* Information and Communication Technologies, *KCCQ* Kansas City Cardiomyopathy Questionnaire, *KOSS* Korean Occupational Stress Scale, *MBCPM* Mindfulness-based chronic pain management, *MBCT* Mindfulness-based cognitive therapy, *MBI* Mindfulness-based interventions, *MCS* Mental component summary, *MDs* Medical doctors, *MDD* Major Depressive Disorder, *MFIS* Modified Fatigue Impact Scale, *MOSS* Medical Outcomes Study Sleep scale, *MS* Multiple sclerosis, *MSD* Musculoskeletal disorders, *MSPSS* Multidimensional Scale of Perceived Social Support, *MSQOL-*54 Multiple sclerosis quality of life-54, *N/A* Not applicable, *NRS* Numeric Rating Scale, *PANAS* Positive and Negative Affect Scale, *PAQ* Pain Awareness Questionnaire, *PCL-C* PTSD Checklist-Civilian Version, *PCS* Physical component summary, *PD* Parkinson’s Disease, *PHQ-*8 Personal Health Questionnaire, *POV* Point of view, *PRISM* The Pictorial Representation of Illness and Self Measure, *PSEQ* Pain self-efficacy questionnaire, *PSS* Perceived Stress Scale, *PSQI* Pittsburgh Sleep Quality Index, *PTSD* Post-traumatic stress disorder, *QoL* Quality of life, *RCT* Randomized Control Trial, *RDAS* Revised Dyadic Adjustment Scale, *RSES* Response to Stressful Experiences Scale, *RULA* Rapid Upper Limb Assessment, *SCD* Sickle-cell disease, *SCS* Self-compassion scale, *SCID* Structured Clinical Interview for DSM Disorders, *SCL*-20 Hopkins Symptoms Checklist, *SF*-36 Short-form 36 Health Survey. *SLC*-20 Hopkins Symptom Checklist, *SNQ* Standard Nordic Questionnaire, *STAI* State Trait Anxiety Inventory, *SUS* System Usability Scale, *TAU* Treatment as usual, *UES* Upper extremity symptoms, *VA* Veteran’s Affairs, *WRMSD* Work-related musculoskeletal disorders

**Table 3:** Participant characteristics of the included interventional studies investing CAIMs delivered via telemedicine (*n* = 56)

Author, Year	Article Title	Intervention Sample Size	Control Sample Size	Intervention Drop-Out	Control Drop-Out	% Female	Mean Age (SD) or Median Age (IQR) in Years	Health Related Condition/Population
Addington et al., 2018 [25]	Convenient and Live Movement (CALM) for Women Undergoing Breast Cancer Treatment: Challenges and Recommendations for Internet-Based Yoga Research	*n* = 6	Not applicable	*n* = 2	Not applicable	100%	59 (12.7)	Cancer
Berman et al., 2009 [27]	The Effectiveness of an Online Mind–Body Intervention for Older Adults with Chronic Pain	*n* = 52	*n* = 37	*n* = 11	*n* = 0	87.80%	65.8	Chronic pain
Bombardier et al., 2013 [28]	Telephone-Based Physical Activity Counseling for Major Depression in People with Multiple Sclerosis	*n* = 44	*n* = 48	*n* = 0	*n* = 0	86%	48	MDD and MS
Cavalera et al., 2019 [29]	Online Meditation Training for People with Multiple Sclerosis: A Randomized Controlled Trial	*n* = 54	*n* = 67	*n* = 8	n = 23	64.40%	Intervention: 42.26 (8.35), control: 43.19 (9.02)	MS
Cheung et al., 2018 [30]	Usability Testing of a Smartphone Application for Delivering Qigong Training	Pilot: *n* = 14, main test: *n* = 100	Not applicable	*n* = 0	Not applicable	Pilot: 71%, main test: 74%	Pilot: 32.3 (8.7), main: 36.15 (13.525)	Cantonese or Putonghua speaking adults who owned a smartphone
Davis et al., 2015 [31]	Mindfulness Training for Smokers via Web-Based Video Instruction with Phone Support: A Prospective Observational Study	*n* = 26	Not applicable	*n* = 6	Not applicable	57.70%	40.5 (13.48)	Smokers
Dimitropoulous et al., 2017 [32]	Evaluating the Feasibility of a Play-Based Telehealth Intervention Program for Children with Prader-Willi Syndrome	*n* = 10	Not applicable	*n* = 2	Not applicable	30%	Not available	Prader-Willi syndrome
Donesky et al., 2017 [33]	Evaluation of the Feasibility of a Home-Based TeleYoga Intervention in Participants with Both Chronic Obstructive Pulmonary Disease and Heart Failure	*n* = 7	*n* = 8	*n* = 1	*n* = 2	66%	Intervention: 73 (14.3), control: 70.5 (2.7)	COPD and HF
Ezenwa et al., 2016 [34]	A Randomized Controlled Pilot Study Feasibility of a Tablet-Based Guided Audio-Visual Relaxation Intervention for Reducing Stress and Pain in Adults with Sickle Cell Disease	*n* = 15	*n* = 13	*n* = 3	*n* = 1	70%	31.70 (10.2)	Sickle cell disease
Ferraris et al., 2020 [35]	Use of Remote Monitoring by E-mail for Long-Term Management of the Classic Ketogenic Diet	*n* = 34	Not applicable	*n* = 3	Not applicable	53%	7.5 (IQR 4.0–10.00)	Drug-resistant epilepsy or GLUT-1-DS
Freeman et al., 2014 [36]	A Randomized Trial Comparing Live and Telemedicine Deliveries of an Imagery-Based Behavioral Intervention for Breast Cancer Survivors: Reducing Symptoms and Barriers to Care	*n* = 23	*n* = 47	*n* = 4	*n* = 4	100%	Intervention: 55.57 (9.88), control: 55.28 (7.90)	Cancer survivors
Gardner-Nix et al., 2014 [37]	Exploring the Effectiveness of a Mindfulness-Based Chronic Pain Management Course Delivered Simultaneously to On-Site and Off-Site Patients Using Telemedicine	*n* = 60	*n* = 59	*n* = 0	*n* = 0	75%	52	Chronic pain patients
Golebowicz et al., 2015 [38]	Efficacy of a Telerehabilitation Intervention Programme Using Biofeedback Among Computer Operators	*n* = 12	Not applicable	*n* = 0	Not applicable	50%	34.25 (8.80)	Work-related musculoskeletal disorders
Guétin et al., 2016 [40]	Smartphone-based Music Listening to Reduce Pain and Anxiety Before Coronarography: A Focus on Sex Differences	*n* = 35	Not applicable	*n* = 0	Not applicable	48.50%	61.26 (11.64)	Management of pain and anxiety in coronarography patients
Hansen et al., 2015 [41]	A Feasibility Pilot Study on the Use of Complementary Therapies Delivered via Mobile Technologies on Icelandic Surgical Patients' Reports of Anxiety, Pain, and Self-efficacy in Healing	Total: *n* = 81, audio-relaxation: *n* = 25, music: *n* = 25, nature video, no music: *n* = 16, nature video with music: *n* = 15	n = 24	*n* = 0	*n* = 0	80% (Note: this was calculated for the purpose of this review)	Audio-relaxation: 45.2 (13.4), music: 46 (15), nature video, no music: 44.60 (16.5), nature video with music: 43.90 (13.5)	Same day surgery patients
Hasan et al., 2019 [42]	Skye Hypnotherapy for Irritable Bowel Syndrome: Effectiveness and Comparison with Face-to-Face Treatment	*n* = 20	Not applicable	*n* = 0	Not applicable	75%	38.40	Irritable bowel syndrome
Hernandez et al., 2018 [43]	Feasibility of an Internet-Based Positive Psychological Intervention for Hemodialysis Patients with Symptoms of Depression	*n* = 14	Not applicable	*n* = 2	Not applicable	50%	57.43 (12.12)	Hemodialysis patients with symptoms of depression
Horneber et al., 2018 [44]	Addressing Unmet Information Needs: Results of a Clinician-Led Consultation Service About Complementary and Alternative Medicine for Cancer Patients and Their Relatives	Total: *n* = 5259, patients: *n* = 3009, caregivers: *n* = 2260	Not applicable	*n* = 0	Not applicable	Total: 65.40%, patients: 64.10%, caregivers: 66.80%	Total: 55 (13), patients: 56 (12), caregivers: 49 (15)	Cancer patients and relatives
Houweling et al., 2015 [45]	First-Contact Care with a Medical vs Chiropractic Provider After Consultation with a Swiss Telemedicine Provider: Comparison of Outcomes, Patient Satisfaction, and Health Care Costs in Spinal, Hip, and Shoulder Pain Patients	*n* = 316	*n* = 403	*n* = 0	*n* = 0	40.80%	Intervention: 41.30 (12.93), Control: 45.70 (13.87)	Spinal, hip, and shoulder pain patients
Huberty et al., 2017 [47]	Experiences of Women Who Participated in a Beta-Test for an Online-Streamed Yoga Intervention After a Stillbirth	*n* = 74	Not applicable	*n *= 22	Not applicable	100%	33.73 (4.38)	Women after a stillbirth
Hucker et al., 2014 [48]	An Online, Mindfulness-Based, Cognitive-Behavioral Therapy for Female Sexual Difficulties: Impact on Relationship Functioning	*n* = 26	*n* = 31	*n* = 20	*n* = 6	100%	Intervention: 33.31 (7.4), control: 31.94 (5.17)	Female sexual difficulties
Kahn et al., 2016 [49]	Post-9/11 Veterans and Their Partners Improve Mental Health Outcomes With a Self-Directed Mobile and Web-Based Wellness Training Program: A Randomized Controlled Trial	*n* = 240	*n* = 80	*n* = 4	*n* = 0	Veterans: 19%, partners of veterans: 93%	Not available	Post-9/11 veterans
Kemper et al., 2017 [50]	Online Training in Mind–Body Therapies: Different Doses, Long-Term Outcomes	*n* = 149	Not applicable	*n* = 0	Not applicable	79.80%	Not available	Outcomes for health professionals
Kim et al., 2020 [51]	Telemedicine Center of Korean Medicine for Treating Patients with COVID-19: A Retrospective Analysis	*n* = 1742	Not applicable	*n* = 0	Not applicable	76.10%	Not available	Hospital patients with COVID-19
Krampe, & Musterman, 2013 [53]	Shall We Skype Dance? Connecting Nursing Students with Older Adults via Skype for Dance-Based Therapy	*n* = 10	Not applicable	*n* = 4	Not applicable	60%	Not available	Older adults in an assisted living facility
Krout et al., 2010 [54]	Designing, Piloting, and Evaluating an On-Line Collaborative Song-writing Environment and Protocol Using Skype Telecommunication Technology: Perceptions of Music Therapy Student Participants	*n* = 4	Not applicable	*n* = 0	Not applicable	75%	Not available	Song-writing students
Kubo et al., 2019 [55]	A Randomized Controlled Trial of mHealth Mindfulness Intervention for Cancer Patients and Informal Cancer Caregivers: A Feasibility Study Within an Integrated Health Care Delivery System	Patient: *n* = 54, caregiver: *n* = 17	Patient: *n* = 43,caregiver: *n* = 14	Patient: *n* = 14, Caregiver: *n* = 4	Patient: *n* = 11, caregiver: *n* = 1	66%	Patient intervention: 59.3 (14.1), caregiver intervention: 57.1 (17.4), Patient control: 56.7 (14.7), Caregiver control: 58.2 (18.6)	Cancer patients and caregivers
Lee et al., 2020 [57]	Long-term Beneficial Effects of an Online Mind–Body Training Program on Stress and Psychological Outcomes in Female Healthcare Providers: A Non-Randomized Controlled Study	*n* = 25	*n* = 31	*n* = 17	*n* = 14	100%	Intervention: 36.20 (8.17), control: 35.00 (6.74)	Female healthcare providers
Lester et al., 2019 [58]	Virtual Mind–Body Treatment for Geographically Diverse Youth with Neurofibromatosis: A Pilot Randomized Controlled Trial	*n* = 27	*n* = 24	*n* = 0	*n* = 0	41.10%	Intervention: 14.48 (1.34), control: 14.26 (1.70)	Youth with neurofibromatosis
Mussman, 2016 [59]	A Mixed-Methods Feasibility Study on the Provision of a Brief Online Yoga Intervention as e-Health for Improving Stress Management: Perceived Stress, Stage of Change for Stress management, and Self-efficacy for Stress Management and Engagement in Yoga	*n* = 14	Not applicable	*n* = 49	Not applicable	85.70%	43.86 (10.52)	Stress
Ondersma et al., 2019 [60]	Feasibility and Acceptability of e-Screening and Brief Intervention and Tailored Text Messaging for Marijuana Use in Pregnancy	*n* = 45	Not applicable	*n* = 0	Not applicable	100%	24.90 (5.2)	Cannabis users during pregnancy
Papadaki et al., 2016 [61]	Employees' Expectations of Internet-Based, Workplace Interventions Promoting the Mediterranean Diet: A Qualitative Study	*n* = 29	Not applicable	*n* = 0	Not applicable	51.70%	42.60 (9.5)	Mediterranean diet in the workplace
Petersen et al., 2017 [62]	An Online Educational Program Improves Pediatric Oncology Nurses' Knowledge, Attitudes, and Spiritual Care Competence	*n* = 112	Not applicable	*n* = 0	*n* = 0	98.20%	Not available	Pediatric oncology nurses
Reilly-Spong et al., 2015 [63]	Telephone-Adapted Mindfulness-Based Stress Reduction (tMBSR) for Patients Awaiting Kidney Transplantation: Trial Design, Rationale and Feasibility	*n* = 32	*n* = 31	*n* = 4	*n* = 4	57.10%	52.80 (11.7)	Patients awaiting kidney transplantation
Rickhi et al., 2015 [64]	Evaluation of a Spirituality Informed e-Mental Health Tool as an Intervention for Major Depressive Disorder in Adolescents and Young Adults—A Randomized Controlled Pilot Trial	*n* = 33	*n* = 29	*n* = 8	*n* = 4	66.67% (Note: this was calculated for the purpose of this review)	Intervention, younger age: 15.30, intervention, older age: 21, control, younger age: 15.20, control, older age: 20.90	Youth with MDD
Rogante et al., 2010 [65]	Electromyographic Audio Biofeedback for Telerehabilitation in Hospital	*n* = 1	Not applicable	Not applicable	Not applicable	Not available	59	A patient with arm impairment following a stroke
Rosmarin et al., 2010 [66]	A Randomized Controlled Evaluation of a Spiritually Integrated Treatment for Subclinical Anxiety in the Jewish community, Delivered via the Internet	*n* = 78	*n* = 47	*n* = 0	*n* = 0	76.60%	41.80 (13.6)	Subclinical anxiety among Jewish community
Rybarczyk et al., 1999 [67]	Comparing Mind–Body Wellness Interventions for Older Adults with Chronic Illness: Classroom Versus Home Instruction	*n* = 115	*n* = 63	*n* = 0	*n* = 0	Classroom: 80.5%, home: 83.6%, control: 81.5%	Classroom: 67.60, home: 61.50, control: 64.70	Older adults with chronic illness
Sarah et al., 2019 [68]	Effect of Telerehabilitation on Long-Term Adherence to Yoga as an Antihypertensive Lifestyle Intervention: Results of a Randomized Controlled Trial	*n* = 115	*n* = 113	*n* = 27	*n* = 26	0%	Intervention: 53.20 (6.0), Control: 53.40 (5.7)	Rehabilitation patients with hypertension
Seidler et al., 2017 [69]	Feasibility and Preliminary Efficacy of a Telerehabilitation Approach to Group Adapted Tango Instruction for People with Parkinson Disease	*n* = 10	*n* = 10	*n* = 3	*n* = 3	55%	Intervention: 68.10 (7.9), control: 68.90 (9.4)	Parkinson’s disease
Selman et al., 2015 [23]	Appropriateness and Acceptability of a Tele-Yoga Intervention for People with Heart Failure and Chronic Obstructive Pulmonary Disease: Qualitative Findings From a Controlled Pilot Study	*n* = 7	*n* = 8	*n* = 1	*n* = 2	75%	71.20 (10.09)	HF and COPD
Shrier et al., 2014 [70]	“Counselor in your pocket”: Youth and Provider Perspectives on a Mobile Motivational Intervention for Marijuana Use	*n* = 20	Not applicable	*n* = 11	Not applicable	62.50%	19.80	Youth who frequently use cannabis, and providers who treat them
Simpson et al., 2002 [71]	Video-Hypnosis—The Provision of Specialized Therapy via Videoconferencing	*n* = 15	Not applicable	*n* = 4	Not applicable	Not available	Not available	Patients with mental health problems who live in remote areas
Singh et al., 2017 [72]	Tele-Health Training of Teachers to Teach a Mindfulness-Based Procedure for Self Management of Aggressive Behavior to Students with Intellectual and Developmental Disabilities	*n* = 3	Not applicable	*n* = 0	Not applicable	0%	10.66	Students with Intellectual and Developmental Disabilities
Stubberud et al., 2020 [73]	Biofeedback Treatment App for Pediatric Migraine: Development and Usability Study	*n* = 10	Not applicable	*n* = 0	Not applicable	30%	15 (1.6)	Pediatric migraine patients
Tan et al., 2013 [74]	Improving Access to Care for Women Veterans Suffering from Chronic Pain and Depression Associated with Trauma	*n* = 34	Not applicable	*n* = 7	Not applicable	100%	49.50 (10)	Female veterans with chronic non-malignant musculoskeletal pain
Thompson et al., 2015 [75]	Expanding the Efficacy of Project UPLIFT: Distance Delivery of Mindfulness-Based Depression Prevention to People with Epilepsy	*n* = 62	*n* = 56	*n* = 10	*n* = 0	65.30%	41.20	Epilepsy
Tkatch et al., 2017 [76]	A Pilot Online Mindfulness Intervention to Decrease Caregiver Burden and Improve Psychological Well-Being	*n* = 40	Not applicable	*n* = 0	Not applicable	80%	71	Community dwelling older adult caregivers
Tucker et al., 2008 [77]	Telephone-Based Diet and Exercise Coaching and a Weight-Loss Supplement Result in Weight and Fat Loss in 120 Men and Women	*n* = 64	*n* = 64	*n* = 11	*n* = 2	50%	43 (9)	Overweight or obese adults
Uebelacker, et al., 2018 [78]	Examining the Feasibility and Acceptability of an Online Yoga Class for Mood Disorders: A MoodNetwork Study	*n* = 56	Not applicable	*n* = 12	Not applicable	93.20%	42.0 (14.62)	Adults with a mood disorder
Vederhus et al., 2020 [79]	Can a Smartphone App for Cannabis Cessation Gain a Broader User Group than Traditional Treatment Services?	*n* = 148	*n* = 102	*n* = 0	*n* = 0	37%	Intervention: 25 (9), control: 25 (8)	Cannabis use disorder patients
Vranceanu et al., 2016 [80]	Mind–Body Therapy via Videoconferencing in Patients with Neurofibromatosis	*n* = 32	*n* = 31	*n* = 0	*n* = 10	73%	Intervention: 42.86 (13.45), control: 39.90 (11.17)	Patients with neurofibromatosis
Wang et al., 2011 [24]	Biomedical Teleacupuncture Between China and Austria Using Heart Rate Variability, Part 1: Poststroke Patients	*n* = 29	Not applicable	*n* = 0	Not applicable	51.70%	64.7 (11.3)	Post-stroke patients
Wang et al., 2016 [81]	The Effects of Music Intervention on Sleep Quality in Community-Dwelling Elderly	*n* = 32	*n* = 32	*n* = 0	*n* = 0	80.90%	69.38 (5.46)	Community-dwelling elderly
Yeh et al., 2013 [82]	Auricular Acupressure Combined with an Internet-Based Intervention or Alone for Primary Dysmenorrhea: A Control Study	*n* = 54	*n* = 53	*n *= 4	*n* = 3	100%	Intervention: 16.94 (1.02), Control: 17.94 (0.84)	Adolescents with primary dysmenorrhea
Zwart et al., 2000 [84]	The Impact of Lay Pastoral Telecare on the Spiritual Well-Being of Church Attenders	*n* = 64	*n* = 63	*n* = 10	*n* = 9	71.60%	37.90 (9.84)	Spiritual well-being in church attenders

Abbreviations: *COPD* Chronic Obstructive Pulmonary Disease, *GLUT*1-*DS* Glucose transporter type 1 deficiency syndrome, *HF* Heart failure, *IQR* Interquartile range, *MDD* Major Depressive Disorder, *MS* Multiple sclerosis, *N/A* Not applicable, *SD* Standard deviation

Armin et al., 2020 [26] was excluded as the sub-groups could not be broken down. Green et al., 2020, Hu et al., 2013, [46] Kwon et al., 2020, [56] and Zini et al., 2018 [83] were excluded as they were non-interventional studies. Krampe et al., 2016 [52] was excluded as the sample described was the same as the Krampe & Musterman 2013 study [53]

**Table 4:** Major telemedicine findings and type of research in the included studies investigating CAIMs used in the context of telemedicine

First Author and Year	Major Telemedicine Finding
Addington et al., 2018 [25]	Internet delivery may increase patients’ access to cancer-adapted yoga classes, but cancer-related and technological barriers remain
Armin et al., 2020 [26]	Research indicates the need to build on smokers’ understandings of CAM techniques, such as meditation or mindfulness, to make guided imagery an appealing tool for smoking cessation
Berman et al., 2009 [27]	The study suggests that the Internet can be an efficient mode for delivering self-care education to older adults with chronic pain and has potential benefits that complement clinical care
Bombardier et al., 2013 [28]	Telephone-based physical activity promotion represents a promising approach to treating MDD in MS. Further research is warranted on ways to bolster the impact of the intervention and on mediators of the treatment effect
Cavalera et al., 2019 [29]	An online mindfulness based intervention could be an effective psychological treatment for the promotion of well-being in MS in short-term. However, the lack of lasting effects requires the development of new strategies to support long-term changes
Cheung et al., 2018 [30]	The majority of users found the *Qigong App* pleasant, user friendly, and useful for learning qigong. Participants indicated positive ratings for the items assessing usability and acceptance of the *App*
Davis et al., 2015 [31]	Results suggest that Mindfulness Training for Smokers can be provided via web-based video instruction with phone support and yield reasonable participant engagement on intervention practices and that intervention efficacy and mechanism of effect deserve further study
Dimitropoulos et al., 2017 [32]	These findings support using telehealth in rare disorders and delivering intervention directly to children with developmental delays through this modality
Donesky et al., 2017 [33]	Tele-Yoga is an acceptable and appropriate intervention in people with HF and COPD and further research is warranted to refine the technology used in its delivery
Ezenwa et al., 2016 [34]	The tablet-based guided relaxation intervention shows promise for reducing sickle cell pain and warrants a larger efficacy trial
Ferraris et al., 2020 [35]	Constant remote monitoring by e-mail could be a feasible and effective way for a better cKD management
Freeman et al., 2014 [36]	There were no significant differences between live delivery and tele delivery, suggesting telemedicine delivered ERL intervention may represent an effective and viable option for cancer survivors in remote areas
Gardner-Nix et al., 2014 [37]	The present study lends support for the effectiveness of mind–body interventions in improving mental health and suffering in chronic pain sufferers seeking help in tertiary pain clinic settings. It also provides evidence for the usefulness of a mindfulness-based program modified specifically for the chronic pain population and supports its delivery through telemedicine
Golebowicz et al., 2015 [38]	This study found that it was feasible and partially effective to integrate a tele-biofeedback ergonomic intervention programme for computer operators suffering from WRMSD
Green et al., 2020 [39]	It was possible to quickly implement real time video conferencing and other forms of telehealth for chiropractic services at 2 worksite health centers
Guétin et al., 2016 [40]	The smartphone-based Music Care application is an easy-to-use tool to reduce anxiety in patients undergoing coronarography
Hansen, 2015 [41]	The feasibility of the implementation of novel interventions of Nature Video and Music (NVAM) and NVAM adds to clinical practice and the complementary therapy literature
Hasan et al., 2019 [42]	Skype hypnotherapy is effective but slightly less so than face-to-face treatment. However, many patients would have been unable to access treatment without the Skype option
Hernandez et al., 2018 [43]	An innovative Internet-based positive psychological intervention represents a feasible and useful therapeutic option for hemodialysis patients with depressive symptoms
Horneber et al., 2018 [44]	Consulting about CAM addresses important unmet needs from cancer patients and their relatives
Houweling et al., 2015 [45]	Spinal, hip, and shoulder pain patients had clinically similar pain relief, greater satisfaction levels, and lower overall cost if they initiated care with Chiropractors, when compared with those who initiated care with Medical Doctors
Hu et al., 2013 [46]	The result shows that the contribution made by the cloud system to the Traditional Chinese Medicine service is multi-dimensional: cost-effective, environment-protective, and performance-enhancing
Huberty et al., 2017 [47]	Overall women who completed three or more weeks of the online intervention were satisfied with online yoga as a means of delivering an intervention after a baby’s death. Women perceived the online yoga as beneficial to both their mental and physical health, ability to be more aware and calm, and self-care. Women reported barriers shared by other middle-aged women and/or women of live births (e.g., time, motivation, family responsibilities). All but one would recommend it to other mothers of stillborn children
Hucker et al., 2014 [48]	The intervention resulted in significant improvement in sexual intimacy and communication, and in emotional intimacy for study group 1. Most improvements were maintained at follow-up
Kahn et al., 2016 [49]	Both veterans and partners were able to learn and make sustained use of a range of wellness practices taught in the MR program
Kemper et al., 2017 [50]	Online training in mind–body therapies is associated with changes in self-reported behavior one year later; increasing doses of training are associated with more frequent practice which is associated with less stress, burnout, and missing work, and higher levels of mindfulness, resilience, and confidence in providing compassionate care
Kim et al., 2020 [51]	Telemedicine care also has shown that even with patient's residence transition, medical care can be continued without pause
Krampe et al., 2016 [52]	Overall, Fuze is a feasible, engaging, and satisfying approach for dance-based therapy, with better audio and visual performance than Skype. The use of synchronous technology to provide therapeutic activities for older adults is an area of research and exploration that appears to have great potential
Krampe, & Musterman, 2013 [53]	With the dedicated efforts of a few key persons, Skype can be an option for the future to connect nurses as well as nursing students with patients
Krout et al., 2010 [54]	Recommendations and suggestions were made by students on how to improve the set-up of music therapy telehealth environment for song-writing sessions
Kubo et al., 2019 [55]	It is feasible to conduct a randomized trial of an mHealth mindfulness intervention for cancer patients and their informal caregivers
Kwon et al., 2020 [56]	Created a manual to introduce insights into the development of mental health interventions for COVID-19
Lee et al., 2020 [57]	Through a convenient, affordable, and easily accessible online format, mindfulness based therapy may provide cost-effective solutions for employees at worksites
Lester et al., 2019 [58]	The mind–body video conferencing was well accepted, highly feasible and resulted in sustained improvement in QoL, demonstrating adolescents are receptive to and benefit from learning resiliency skills in groups via live video
Mussman, 2016 [59]	The pilot study’s findings support the feasibility of providing online four-week yoga e-health intervention
Ondersma et al., 2019 [60]	These two high-reach intervention elements showed strong feasibility and modest to high acceptability
Papadaki et al., 2016 [61]	An Internet-based, workplace MedDiet intervention should address adherence barriers, utilize a tailored approach to setting and reviewing goals, and activate social support to facilitate adherence. These findings indicate that the MedDiet is suitable for those in non-Mediterranean areas
Petersen et al., 2017 [62]	Online spiritual care educational programs may exert a lasting impact on nurses’ attitudes toward and knowledge of spiritual care and their competence to provide spiritual care to children with cancer at the end of life
Reilly-Spong et al., 2015 [63]	Telephone-Adapted Mindfulness-Based Stress Reduction is an accessible intervention that may be useful to people with a wide spectrum of health conditions
Rickhi et al., 2015 [64]	The results of the e-mental health LEAP Project pilot trial suggest that it is an effective, online intervention for youth ages 13 to 24 with mild to moderate major depressive disorder with various life situations and in a limited way on spiritual well-being and self-concept
Rogante et al., 2010 [65]	Wireless technology such as surface electromyography with biofeedback allows a reduction in complexity of tasks required of patients with arm impairments
Rosmarin et al., 2010 [66]	It is important to incorporate spiritual content into treatment to help facilitate the delivery of psychotherapy to religious individuals
Rybarczyk et al., 1999 [67]	A lower cost, more accessible home study version of a mind–body wellness program can be an effective alternative to classroom instruction
Sarah et al., 2019 [68]	Telerehabilitation significantly improves yoga adherence maintaining achieved health benefits in the long term
Seidler et al., 2017 [69]	This pilot study suggests a telerehabilitation approach to group tango class for people with Parkinson Disease is feasible and may have similar outcomes to in-person instruction
Selman et al., 2015 [23]	Tele-Yoga is an acceptable and appropriate intervention in people with HF and COPD and further research is warranted to refine the technology used in its delivery
Shrier et al., 2014 [70]	Results suggest that mobile technology is a promising tool for brief interventions to reduce youth cannabis use and warrants further development
Simpson et al., 2002 [71]	Hypnosis can be provided successfully via videoconferencing
Singh et al., 2017 [72]	Tele-health may be an effective approach to providing training and therapy to caregivers in remote locations that cannot readily access specialist services
Stubberud et al., 2020 [73]	An app for young migraine sufferers to receive therapist-independent biofeedback was created. The app has undergone usability and feasibility testing, and is now ready for clinical trials
Tan et al., 2013 [74]	It is feasible to provide treatment to women veterans living in rural areas by utilizing video-teleconferencing technology between larger VA medical centers and facilities at CBOCs in more rural settings
Thompson et al., 2015 [75]	Distance delivery of group MBCT can prevent episodes of MDD, reduce symptoms of depression, and increase life satisfaction in people with epilepsy
Tkatch et al., 2017 [76]	Community-dwelling older adults would successfully engage in an online mindfulness intervention. Retention and participation rates were high with over 50% completing the program. Findings related to the second goal of this study demonstrated that an online mindfulness meditation intervention could positively influence caregiver burden, quality of life, and psychological well-being
Tucker et al., 2008 [77]	Adults can be educated and motivated via telephone to change behaviors leading to weight loss, and a weight-loss supplement can be included to increase success
Uebelacker, et al., 2018 [78]	These preliminary data support the utility of online yoga tailored specifically for people with mood disorders as a possible adjunctive intervention that warrants further investigation
Vederhus et al., 2020 [79]	The app can be an alternative for those who are not yet prepared to seek treatment in formal healthcare services
Vranceanu et al., 2016 [80]	For patients with neurofibromatosis, a mind–body program is superior to an attention placebo control in improving QoL
Wang et al., 2011 [24]	It could be demonstrated that teleacupuncture between China/Harbin and Austria/Graz over a distance of about 8,500 km is no longer a future vision; it has become reality
Wang et al., 2016 [81]	Music is a safe and effective nonpharmacological intervention for improving the sleep quality of community-dwelling elderly people, especially in improving sleep latency, sleep efficiency, and daytime dysfunction
Yeh et al., 2013 [82]	Auricular acupressure combined with interactive Internet instruction is better than auricular acupuncture alone in improving self-care behaviors
Zini et al., 2018 [83]	An mHealth application for training and empowering patients in managing KD can act as a bridge connecting patients with the health care staff for coaching and monitoring purposes
Zwart et al., 2000 [84]	The use of telephone by lay pastoral caregivers can be a means of promoting interpersonal support and enhancement of spiritual well-being within a church congregation

Abbreviations: *CAIM* Complementary, Alternative, and Integrative Medicine, *CAM* Complementary and Alternative Medicine, *CBOC* Community-based outpatient centre, *cKD* Classic ketogenic diet, *COPD* Chronic obstructive pulmonary disease, *HF* Heart failure, *MDD* Major depressive disorder, *MR* Mindfulness reduction, *MS* Multiple sclerosis, *QoL* Quality of life, *RCT* Randomized controlled trial, *VA* Veteran’s affairs, *WRMSD* Work-related musculoskeletal disorders

### CAIM characteristics

Of the 62 articles included, the distribution of CAIMs discussed were as follows: mindfulness training (*n* = 11), mind–body exercise (*n* = 7), yoga (*n* = 7), biofeedback (*n* = 4), music therapy (*n* = 4), spiritual care (*n* = 4), dance therapy (*n* = 3), cannabis (*n* = 3), chiropractic manipulation (*n* = 2), guided imagery (*n* = 2), hypnosis (*n* = 2), ketogenic diet (*n* = 2), acupuncture (*n* = 1), auricular acupressure (*n* = 1), Chinese medicine (*n* = 1), exercise (*n* = 1), qigong (*n* = 1), herbal medicine (*n* = 1), meditation (*n* = 1), Mediterranean diet (*n* = 1), play-based therapy (*n* = 1), and vitamin B weight loss (*n* = 1).

### Telemedicine characteristics

Of the 62 articles included, the telemedicine tools used were as follows: videoconferencing (*n* = 16), mobile application (*n* = 7), web- or mobile-based application (*n* = 2), videos (*n* = 10), websites (*n* = 7), telephone (*n* = 7), database/cloud system (*n* = 1), telemedicine centre (*n* = 1), teleconference (*n* = 1), telephone and video (*n* = 1), e-mail (*n* = 1), remote tele-biofeedback (*n* = 1), social media platform (*n* = 1), telephone and portable electromyograph (*n* = 1), videos and chat group (*n* = 1), text messaging (*n* = 1), telephone and videoconferencing (*n* = 1), telephone and mp3 audio (*n* = 1), and website and videoconferencing (*n* = 1).

### Findings from thematic analysis

In total, three main themes emerged from our analysis and are described below.

### Theme 1: Practitioner view of CAIM telemedicine

#### Feasibility of CAIM telemedicine interventions

Overall, practitioners found it feasible to deliver traditionally in-person CAIM interventions through a telemedicine approach (*n* = 26) [24, 25, 27, 31–35, 38–40, 42, 43, 51, 52, 54, 55, 58–60, 63, 68, 69, 71, 72, 74, 76, 80, 83, 85]. Sufficient technology exists to meet the delivery needs of a great number of heterogeneous CAIM interventions. For example, Skype as a videoconferencing platform could be effectively used for hypnotherapy [42], but also for mind–body therapy [80]. Other technologies such as telephones, internet websites, smartphone applications, virtual-reality technology, and even specialized cloud platforms were successfully tailored to the goals of particular CAIM interventions and targeted towards a diverse range of patient populations including older adults [27]. Practitioners found it feasible to implement physical activities such as dance and yoga virtually [25, 59, 68], but also found it was possible to administer more complex CAIM interventions such as hypnosis therapy, or the virtual management and treatment of patients with COVID-19 [51, 71].

The feasibility of the intervention itself was comparable, and in some cases, superior to in-person delivery. One study found that interest in participation and feasibility of a Skype mind–body therapy was superior compared to an in-person pilot test of the same intervention [43], while another study found increased scheduling flexibility and subsequently, greater participation in the telemedicine intervention compared to in-person care delivery [80]. Telemedicine approaches to CAIM were also more inclusive for participants who would usually have been unable to participate due to cost barriers, or travel difficulties such as urinary incontinence [42].

#### High acceptability and satisfaction of CAIM telemedicine interventions

Practitioners readily accepted and reported favourable attitudes towards telemedicine approaches to CAIM (*n* = 21) [23, 39, 41, 45, 46, 50–57, 61, 63–66, 68, 70, 73]. Practitioners did not have major concerns regarding ease of use, appeal to target population, or efficacy of telemedicine CAIM interventions. This held true across the various populations included in this review. For example, clinicians in a cannabis reduction intervention did not have concerns about confidentiality, or application of mobile device technology [70]. Another telephone-adapted delivery format for a mindfulness-based stress reduction was perceived by practitioners as “very positive” [63]. In a dance-therapy session for older adults, student nurse leaders expressed high interest and enjoyment in intervention delivery among study participants [52]. Moreover, practitioners involved with a study by Green et al. [39] found that telehealth enabled continuity of care with patients and was therefore a “valuable” tool.

A common view was that telemedicine is valuable to improve the efficiency of medical resource use, through reducing wait times for patients [51], improving hospital-bed shortage problems [51], and reducing the workload burden of healthcare staff [45, 46, 50, 73]. Practitioners were also satisfied with the potential to lower healthcare delivery costs [46, 55, 57, 63, 66, 73], in one case by up to 75% [63]. Practitioners believed telemedicine delivery of CAIM had a high potential for wider scalability in the healthcare system [63, 64, 66, 73]. Although, some studies expressed barriers such as a lack of a tailored approach to goal setting in an internet-based workplace intervention promoting a Mediterranean diet [61], and poor software and hardware usability of an electromyographic audio biofeedback program for telerehabilitation [65].

#### Health and well-being improvements

Practitioners found that CAIM interventions delivered using telemedicine resulted in health and well-being improvements across a variety of patient populations, comparable to improvements observed in in-person delivery modes (*n* = 35) [23, 24, 27–29, 31, 33, 36–38, 40, 41, 43, 45, 47–50, 55, 58, 64, 66–69, 72, 74–78, 80–82, 84]. This applied not only to physical patient health [77], but also to quality of life [76], mental [31, 40, 43, 75] and spiritual [64] health, and aspects of personality such as self-concept and self-esteem [64]. The improvement in health was observed across all age groups, from children and adolescents [64], to older adults [67]. Moreover, the improvements to health manifested across a diverse range of patient groups, including veterans, cancer patients, and individuals with chronic illness. Many of these changes were clinically meaningful, having positively impacted the course of the illness or resulted in visible improvements from the perspective of both patients and clinicians [64, 66, 67]. Positive health changes often persisted longitudinally at various follow-up periods, indicating that telemedicine interventions can produce persistent health benefits [28, 43, 48–50, 57, 58, 62, 64, 68, 74, 75, 80, 81]. In some cases, health benefits did not remain at follow-up [29], or longitudinal assessment was not reported.

### Theme 2: Patient view of CAIM telemedicine

#### The patient-practitioner relationship

Patients felt it was challenging to form meaningful connections with CAIM practitioners employing telemedicine alternatives (*n* = 10) [23, 25, 26, 32, 33, 44, 47, 52, 71, 78, 79]. Study participants reported a lack of understanding of the role of the practitioner, difficulty following along with remote-based interventions, and lack of sufficient feedback on their performance from practitioners. For example, participants involved in yoga interventions through video-conferencing technologies identified challenges such as having to continuously “readjust screens,” difficulty “learning and doing poses simultaneously,” a lack of instructor feedback in real-time, and an inability to “bond” with the instructor [25, 47, 78]. In telephone-based coaching interventions, participants seemed to be unclear of the role of coaches, and found it “difficult to develop a relationship with or trust a stranger on the phone” [26, 44]. Furthermore, according to participants, CAIM interventionists may misinterpret their needs particularly when employing audio-visual or phone-based telemedicine technology [25, 26, 29, 41, 47, 67, 71, 78, 85], for reasons such as being unable to perceive “subtle expressions” of interest, emotion, or physical comfort [25], or as a consequence of ineffective communication between practitioners and participants through digital platforms [47, 78].

#### The impact of existing chronic health conditions and morbidities on intervention outcomes

Complex or chronic conditions, as well as multimorbidity, was found to negatively impact participation, patient safety, or retention of patients in CAIM interventions delivered through telemedicine (*n* = 12) [23, 25, 32–34, 37, 43, 47, 52, 55, 63, 69]. In particular, the presence of these types of health conditions were associated with various functional and mobility limitations such as breathing problems and fatigue, which served as a barrier to participation [23, 47, 63]. For example, some individuals with cancer found it difficult to participate in virtual yoga training due to “[cancer] treatment-related fatigue,” and cancer-related overwhelmingness and forgetfulness [25]. Individuals with chronic pain found that their condition interfered with their ability to attend virtual mindfulness-based classes as part of an intervention [37]. However, this issue was acknowledged and the program was lengthened to suit their needs [37]. Other studies noted that attrition was often due to deteriorating health, or health-related responsibilities (e.g., surgery) [33, 43, 55, 63].

#### The benefit of telemedicine delivery of CAIM for traditionally underserved populations

Participants most frequently cited CAIM interventions administered through a telemedicine approach as an accessible alternative to in-person care, that leads to improved health outcomes without any salient consequences (*n* = 21) [25, 27, 28, 30, 32, 37–39, 42, 45–47, 51, 55, 57, 63, 69–71, 74, 76]. Virtual care delivery appeared to expand access to care particularly for rural populations [74], or those with chronic health conditions that prevented them from travelling long distances. Many of the included studies also engaged populations that are often neglected such as racial or ethnic minorities [26, 60], or women veterans [74]. Evaluations and feedback were overwhelmingly positive and in support of these health interventions, noting improved accessibility in receiving CAIM in the comfort of their own homes [32, 43]. Previously identified barriers to participation such as high travel costs [28, 32, 42, 45, 63], inability to travel [28, 42, 63, 69], time conflicts [28, 30, 63], and reluctance to participate in a group or associate with other frail individuals [27], among others, were overcome.

### Theme 3: The technological impacts of CAIM via telemedicine

Overall, technological issues did not appear to impede the success of CAIM delivered via telemedicine. However, some participants did believe that technological difficulties were a hindrance. Broadly, issues included degradation of audio and visual quality, limited access to the necessary devices, complex user interface in applications, and troubles with downloading CAIM intervention content, which are all necessary components in successful telecommunication delivery of CAIM (*n* = 14) [23, 25–27, 29, 30, 32, 33, 42, 43, 46, 47, 54, 72]. For example, an unstable internet connection, especially in rural areas, made it difficult to attend or follow along during CAIM sessions [23, 72]. Even when participants did connect to the telemedicine platform being used, freezing of the video stream or inconsistent audio made it difficult to engage and maximally benefit from the intervention [23, 32, 54]. Consequently, some participants believed the technological difficulties prevented them from gaining the “full benefit of the teacher’s feedback and interaction” [33]. In some cases, the technological difficulty meant that the therapeutic session had to be rescheduled [32]. Other types of technological barriers included font and video screen sizes in a mobile app study [30]. In contrast, practitioners did not generally find that technological difficulties were a significant barrier to the feasibility of intervention delivery, reporting that issues were infrequent [29, 32, 63, 74], and quickly and easily resolved when they did occur [25].

## Discussion

The purpose of this review was to synthesize the literature on telemedicine utilised in the context of CAIM. To our knowledge, this is the first study to explore this field using a systematic search of peer-reviewed and grey literature to inform practice and future areas of research. Overall, CAIM interventions offered through telemedicine approaches are comparable to face-to-face interventions across dimensions of feasibility, clinical efficacy, and patient and provider satisfaction. The presence of complex or chronic health conditions such as cancer, as well as technological difficulties were reported as barriers to patient participation and satisfaction.

The results of our study reveal that telemedicine strategies to deliver CAIM are diverse, including videoconferencing, telephone, mobile applications, email, and cloud platforms. There is also great heterogeneity in the target populations of these interventions. This aligns with previous findings that telemedicine approaches can be effective for populations with diverse physical, mental, and emotional health-care needs [86–88].

Both practitioners and patients overwhelmingly found that telemedicine delivery of CAIM was feasible and acceptable. Practitioners perceived telemedicine as a valuable, cost-effective tool with potential for wider scalability [31, 43, 47, 55]. Furthermore, statistically significant, and clinically meaningful improvements in health outcomes were noted by both patients and practitioners. This reflects evidence telemedicine is found to comparable to face-to-face care in terms of feasibility and clinical-effectiveness [85, 89–92]. Practitioners also cite time savings after implementing telemedicine, due to a reduction in “downtime and inefficiencies” [93]. For many families, telemedicine delivery reduced cost and transportation barriers, increasing access to care. A recent review indicates that telemedicine advancements have improved access to care for a wide range of clinical conditions, and has addressed geographical barriers to care, although social barriers still lack attention [94].

Both patients and providers appear to be highly satisfied with telemedicine delivery of CAIM, citing that technical difficulties that arose in the intervention delivery were quickly and easily resolved. However, more patients noted technological difficulties that interfered with their participation and satisfaction with the intervention relative to providers delivering the intervention. This is in contrast to the literature where patients typically report high satisfaction with telemedicine approaches [95, 96]. One aspect of technological difficulties included a lack of access to the internet or telecommunication devices. This necessitates education to guide patients that may be unfamiliar about the use of various internet and mobile technologies (e.g., videoconferencing platforms, mobile applications), and its benefits in promoting health and well-being [97]. Patients in areas with unstable internet connections such as rural and remote regions also faced additional barriers to participation in telemedicine delivery of CAIM interventions [97, 98]. This may require government action to enhance internet network bandwidth and deploy advanced generations of network technologies to provide the necessary support as telemedicine continues to expand [97]. On the other hand, provider satisfaction with telemedicine has been studied less frequently as there was a lack of evidence found in this review. This is despite provider perspectives being crucial to the expansion of telemedicine [96].

An important barrier identified by patients is that it was more difficult to establish valuable, meaningful connections with care providers virtually compared to face-to-face. Sharing difficult diagnoses and end-of-life conversations are examples of situations where it is challenging to facilitate via telemedicine, and the telemedicine approach cannot replace human connections formed with a face-to-face conversation [99]. This underscores the importance of ensuring that medical practitioners delivering care using telemedicine modes consider the limitations of these approaches. Both patients and providers reflecting on using telemedicine for chronic disease management recommended that the initial patient-provider interaction should be face-to-face, and that patients should see the same provider at follow-up visits [100].

It is also important to consider that participants with chronic health conditions such as cardiovascular disease, cancer, or diabetes may experience condition-specific barriers such as “chemo fatigue,” [25] or functional limitations [101]. Older adults with chronic illness may face additional challenges relating to frailty, vision and hearing loss, and cognitive limitations that have relevance in non-face-to-face interventions [102]. Moreover, elderly patients may not be able to handle large volumes of online information and some older adults may become anxious or annoyed when adding technology to their regular routine [102]. This is supported by past studies, which identified that special populations such as older adults, patients with disabilities (e.g., vision or hearing difficulties), limited mobility, and/or racial and ethnic minorities may face additional barriers in telemedicine delivery of care [97, 98]. Accordingly, training both care providers and patients is paramount for effective delivery of CAIM via telemedicine [97].

### Implications and future directions

Telemedicine models of care have been used for many years, particularly in the United States, but its use has expanded globally during the COVID-19 pandemic [103]. The reviewed literature highlights the potential to deliver CAIM via telemedicine. This study has generated several areas for future research on CAIM delivered using telemedicine.

Further research is required to identify groups that would realize the greatest impact from telemedicine delivery of CAIM. For patients with chronic conditions such as diabetes or cancer, or for older adults, it is important to consider physical or cognitive limitations that may be barriers to successful completion of these interventions. There is also a need to investigate the impact of a more personalized or tailored approach for these high-needs groups with existing illnesses and morbidities [104].

CAIM interventions delivered via telemedicine would benefit from a more holistic evaluation beyond biomedical outcomes. Currently there is a lack of reporting in the literature on how CAIM therapies delivered via telemedicine compare to face-to-face approaches with respect to provider and staff burden, and the experiences of family and friend caregivers [102]. There is also a lack of incorporation of social determinants of health such as socioeconomic status, and race in telemedicine interventions. Evidence indicates that social determinants affect access to telemedicine for groups already suffering from inequities in healthcare access [105]. Accordingly, future telemedicine policy and research should go beyond technological dimensions, and consider social determinants of health [106]. Further, assessment of outcomes at longer follow-up periods is needed to determine whether telemedicine delivery of CAIM is capable of producing sustainable effects [43, 49, 81].

Finally, future work at the intersection of CAIM and telemedicine should identify and evaluate the (in)appropriate use of telemedicine across various CAIM practices. In line with this, more work is needed to examine the facilitators and barriers that providers face in employing telemedicine delivery of CAIM.

### Strengths and limitations

Strengths of this study include adherence to Arksey and O’Malley’s five-stage scoping review framework [12], and the use of a comprehensive systematic search strategy across several bibliographic databases to identify eligible articles. Interpretation of the findings was strengthened by the fact that three authors independently screened, and a total of seven authors extracted, and summarised the findings. There are some limitations to this scoping review. By including studies only written in English, we could be missing important international work. This is especially relevant because CAIM may be practiced more frequently in non-English speaking regions of the world, such as traditional Chinese medicine in China.

Another limitation is that despite the use of a comprehensive search strategy, CAIM is an umbrella term encompassing a broad range of practices and as such, it is possible that not all CAIM therapies were captured in the search. Finally, records outside of those found via bibliographic database searches (e.g., unpublished theses and dissertations) were considered outside of the scope of this review, although we acknowledge that this may have contributed to some relevant literature being missed.

## Conclusions

The present scoping review explored the breadth of the literature on telemedicine used in the context of CAIM. Three main themes were identified: 1) the practitioner view of CAIM telemedicine, 2) the patient view of CAIM telemedicine, and 3) the technological impacts of CAIM telemedicine. These themes highlight the feasibility, acceptability, and satisfaction of CAIM delivered via telemedicine from both a practitioner and patient point of view. Telemedicine approaches increase access to CAIM, and there is high potential for scalability. Patient barriers include chronic illness and morbidities, low technical proficiency, and an inability to form meaningful connections with care providers. Further research is required to mitigate barriers to telemedicine uptake and increase the knowledge of clinicians on topics of CAIM and telemedicine. We recognize that this may take the form of changes to training, management techniques, and health-care policies.

## Data Availability

All relevant data are included in this manuscript.

## Abbreviations

CADTH: Canadian Agency for Drugs and Technologies in Health
CAIM: Complementary, alternative, and integrative medicine
